# ResLysEmbed: a ResNet-based framework for succinylated lysine residue prediction using sequence and language model embeddings

**DOI:** 10.1093/bioadv/vbaf198

**Published:** 2025-08-22

**Authors:** Souvik Ghosh, Md Muhaiminul Islam Nafi, M Saifur Rahman

**Affiliations:** Department of CSE, BUET, Dhaka 1000, Bangladesh; Department of CSE, BRAC University, Dhaka 1212, Bangladesh; Department of CSE, BUET, Dhaka 1000, Bangladesh; Department of CSE, United International University (UIU), Dhaka 1212, Bangladesh; Department of CSE, BUET, Dhaka 1000, Bangladesh

## Abstract

**Motivation:**

Lysine (K) succinylation is a crucial post-translational modification involved in cellular homeostasis and metabolism, and has been linked to several diseases in recent research. Despite its emerging importance, current computational methods are limited in performance for predicting succinylation sites.

**Results:**

We propose ResLysEmbed, a novel ResNet-based architecture that combines traditional word embeddings with per-residue embeddings from protein language models for succinylation site prediction. We also compared multiple protein language models to identify the most effective one for this task. Additionally, we experimented with several deep learning architectures to find the most suitable one for processing word embedding features and developed three hybrid architectures: ConvLysEmbed, InceptLysEmbed, and ResLysEmbed. Among these, ResLysEmbed achieved superior performance with accuracy, MCC, and F1 scores of 0.81, 0.39, 0.40 and 0.72, 0.44, 0.67 on two independent test sets, outperforming existing methods. Furthermore, we applied shapley additive explanations analysis to interpret the influence of each residue within the 33-length window around the target site on the model’s predictions. This analysis helps understand how the sequential position and structural distance of residues from the target site affect their contribution to succinylation prediction.

**Availability:**

The implementation details and code are available at https://github.com/Sheldor7701/ResLysEmbed.

## 1 Introduction

Post-translational modifications (PTMs) play a very crucial role in regulating localization, activities, and interaction with other cellular molecules. Among more than 300 kinds of PTMs, lysine succinylation is relatively new. Succinylation was first reported as a PTM (Zhang *et al.* 2011). As the succinyl group has a larger mass (100 Da), it causes a significant mass change and shifts in charge (from +1 to −1) at the modified lysine residue. These changes heavily affect gene expression, cellular homeostasis, and metabolic pathways (Yang and Gibson 2019).

Recent research on lysine succinylation has linked it to several pathological conditions, including cancer, metabolic disorders, and infectious diseases. For example, studies have associated succinylation with heart diseases (Yang *et al.* 2021), hereditary mitochondrial diseases (Gut *et al.* 2020), and Alzheimer’s disease (Yang *et al.* 2022). Recently, Wang *et al.* (2024) published a study suggesting that succinylation plays a vital role in central nervous system diseases, including stroke, brain tumors, and Alzheimer’s disease. Furthermore, lysine succinylation has been linked to SARS-CoV-2 infection, where succinylated host proteins play a vital role in the virus’s interaction with its host (Liu *et al.* 2022).

Given the emerging importance of succinylation in health and disease, there have been a lot of experimental approaches for its detection (Jin and Wu 2016, Zhou *et al.* 2018, Yuan *et al.* 2019). However, as experimental methods such as high-resolution liquid chromatography-tandem mass spectrometry (LC-MS/MS) are cumbersome, expensive, and time-consuming, various computational methods have been developed for lysine succinylation detection. The first such computational method for this prediction was iSuc-PseAAC (Xu *et al.* 2015b), proposed by Xu *et al.* This procedure was based on a support vector machine (SVM). Later on, many other machine learning–based predictors were built such as SuccFind (Xu *et al.* 2015a), iSuc-PseOpt (Jia *et al.* 2016a), and pSuc-Lys (Jia *et al.* 2016b). However, these studies used a relatively smaller dataset and did not consider the proper distribution of lysine succinylation. Later on, Hasan *et al.* designed a new method, SuccinSite (Hasan *et al.* 2016), along with a new dataset that has been used in many subsequent studies, including ours. Hasan *et al.* crafted two new methods, namely SuccinSite2.0 (Hasan *et al.* 2017) and GPSuc (Hasan and Kurata 2018). Both of these had generic and species-specific succinylation site prediction methods.

Around the same period, several traditional machine learning–based predictors such as PSSM-Suc (Dehzangi *et al.* 2017), Success (López *et al.* 2018), and SSEvol-Suc (Dehzangi *et al.* 2018) were also proposed. These models typically relied on handcrafted features derived from evolutionary profiles and structural properties of amino acids, and employed classifiers like SVMs or ensemble methods to predict succinylation sites. While these contributed valuable insights into the feature representation and modeling of succinylation, the lack of publicly available implementations, differences in dataset versions or preprocessing strategies, and, in some cases, the use of outdated or limited benchmark datasets render these unsuitable for benchmarking against newer methods.

Consequently, deep learning–based methods started to emerge, such as CNN-SuccSite (Huang *et al.* 2019), DeepSuccinylSite (Thapa *et al.* 2020), pSuc-EDBAM (Jia *et al.* 2022), and LMSuccSite (Pokharel *et al.* 2022). CNN-SuccSite used convolutional neural network (CNN)-based architecture along with various feature encoding techniques as input for the task. DeepSuccinylSite (Thapa *et al.* 2020) used the idea of word embedding for the first time and determined the optimal window size with various experiments. Subsequently, LMSuccSite (Pokharel *et al.* 2022) used word embedding (Thapa *et al.* 2020) along with language model embeddings to build an ensemble architecture, which has been quite successful. Along the same time, Jia *et al.* created an ensemble dense block-based method with an attention module named pSuc-EDBAM (Jia *et al.* 2022), and Ahmed *et al.* proposed several CNN bidirectional LSTM-based ensemble architectures (Ahmed *et al.* 2023) based on different biophysical properties as input. Both of these, despite attaining impressive performance, could not outperform LMSuccSite (Pokharel *et al.* 2022). PTM-CMGMS (Li *et al.* 2024) and PTMGPT2 (Shrestha *et al.* 2024), two new models, came out during the development of this manuscript. These are several PTM detecting methods where a fixed architecture is trained with different PTM datasets to create separate PTM prediction models. In PTM-CMGMS (Li *et al.* 2024), structural information is utilized using various feature encoding techniques, whereas in PTMGPT2 (Shrestha *et al.* 2024), a pretrained GPT2-based model PROTGPT2 (Ferruz *et al.* 2022) was fine-tuned for each PTM detection task. However, despite being excellent approaches, both of these methods require quite a lot of computational resources with marginal improvement over the existing predictors in terms of succinylation prediction. Moreover, upon closer inspection on PTMGPT2 (Shrestha *et al.* 2024) for comparison purposes, we found multiple discrepancies in the succinylation dataset, which is elaborately discussed in Section 3.4. Most recently, a cross-species predictor named KD_MultiSucc (Tran *et al.* 2025) was proposed, which combines multiteacher knowledge distillation with word embeddings to balance computational efficiency and predictive accuracy.

Despite recent advances in computational methods for lysine succinylation prediction, several challenges and unexplored areas remain. For example, there has not been any comparative analysis of how well various PLMs perform in this task. In addition, many methods often prioritize performance and overlook interpretability, leaving unidentified insights into the biological relevance of the predictions. Finally, recent predictors like PTMGPT2 (Shrestha *et al.* 2024) need high computational resources while also exhibiting dataset discrepancies. Our study addresses these gaps by developing ResLysEmbed, a novel, efficient, and interpretable architecture for the prediction of lysine succinylation.

Inspired by the success of LMSuccSite (Pokharel *et al.* 2022), we too decided on using protein language model (PLM) embeddings as features. We did a comparative analysis on four PLMs, namely ProtT5 (Elnaggar *et al.* 2022), ESM-650M (Lin *et al.* 2023), ESM-3B (Lin *et al.* 2023), and PTM-Mamba (Peng 2024) to determine which performed the best for this task. We compared these language model embeddings along with word embedding and position-specific scoring matrix (PSSM) using two different feature selection techniques. Additionally, we did a comparative analysis with various architectures to determine which was the most suitable for processing word embedding features. Based on these analyses, we established that the Conv2D architecture proposed in LMSuccSite (Pokharel *et al.* 2022) performed suboptimally for capturing sequential information from word embeddings. Finally, we designed three hybrid architectures referred to as ConvLysEmbed, InceptLysEmbed, and ResLysEmbed. Our final model, ResLysEmbed, outperforms all the state-of-the-art methods, including the recent predictors, with an improvement varying from 2% to 13% on one independent test set across various performance metrics. Additionally, on a separate benchmark test set, ResLysEmbed achieves the highest scores across all core metrics, including accuracy (72.64%), MCC (0.4444), AUROC (0.7889), and F1 score (0.6727), demonstrating superior generalizability and robustness. Moreover, this new architecture has fewer parameters than most existing methods which makes it a computationally efficient and easy-to-use tool for predicting succinylation sites.

Additionally, to interpret our results more robustly, we conducted shapley additive explanations (SHAP) (Lundberg and Lee 2017) analysis on ResLysEmbed. This interpretability analysis helped us understand the relative importance of each residue within the 33-length window around the target lysine site. We identified the key factors that affect a residue’s significance in succinylation prediction by analyzing the contributions of each residue based on both relative sequential position from the target site and structural distance from the target site. The outcome of this analysis reaffirms the biological relevance of our findings, making ResLysEmbed a valuable tool for understanding succinylation’s underlying mechanisms.

In summary, our key contributions are as follows,

- We developed a new ResNet + multilayer perceptron (MLP)-based hybrid model (ResLysEmbed) for lysine succinylation site prediction that outperforms state-of-the-art methods while being relatively simpler and computationally efficient.
- We conducted SHAP analysis to interpret the relative importance of each residue within the 33-length window surrounding the target lysine site which provides insights into the biological relevance of ResLysEmbed.
- We conducted a comparative analysis of different PLM embeddings for lysine succinylation site prediction.
- We also conducted a comparative analysis of different architectures for processing word embeddings and ProtT5 embeddings.
- We developed and evaluated three hybrid architectures: ConvLysEmbed, InceptLysEmbed, and ResLysEmbed.
- We prepared an external test set derived from the dbPTM database and used it to demonstrate the generalizability of our model.

## 2 Methods

### 2.1 Dataset

For our work, we used the same dataset as was used in developing LMSuccSite (Pokharel *et al.* 2022). This dataset was originally developed by Hasan *et al.* (2016) from the UniProtKB/Swiss-Prot (Consortium 2015) database and the NCBI protein sequence database. It contained experimentally verified succinylation sites.

Hasan *et al.* (2016) used CD-HIT(Li and Godzik 2006) with a 30% sequence similarity cut-off to ensure that no sequence shared more than 30% similarity with any other sequence in the dataset. As a result, 5009 succinylation sites from 2322 protein sequences were added to the dataset. Then the dataset was randomly split into training and testing sets. There were 2192 protein sequences in the training set, which included 4750 succinylation sites, and 124 protein sequences with 254 succinylation sites were present in the test set. All other lysine sites were considered negative succinylation sites, which resulted in 50 565 negative succinylation sites in the training dataset and 2977 in the test set. Finally, the training dataset was balanced using random undersampling. This dataset was also used in developing DeepSuccinylSite (Thapa *et al.* 2020), pSuc-EDBAM (Jia *et al.* 2022), PTM-CMGMS (Li *et al.* 2024) (for succinylation), and many other models.

To further enhance our evaluation, we incorporated an additional benchmark dataset derived from the dbPTM database (Chung *et al.* 2025). This newer dataset contains a larger collection of experimentally validated succinylation sites. To ensure non-redundancy with the training data, we applied CD-HIT-2D (Li and Godzik 2006) to remove any sequences from the dbPTM benchmark dataset with high similarity to those in the original training dataset. The final dbPTM benchmark set includes 4120 labeled data points (1901 positive and 2309 negative) from 1815 unique proteins. From here onward, we refer to this dataset as dbPTM1815 for clarity and consistency. The final dbPTM1815 dataset was used for independent model evaluation and the interpretability analysis discussed in Section 2.6. The summary of the datasets is given in Table 1.

#### 2.1.1 Performance metrics

For evaluation, we used several widely accepted metrics for binary classification: accuracy, precision, recall, F1 score, specificity, Matthews correlation coefficient (MCC), area under the receiver operating characteristic curve (AUROC), and area under the precision-recall (PR) curve (AUPRC). These metrics are derived from the standard confusion matrix components: true positives (TP), true negatives (TN), false positives (FP), and false negatives (FN).

- **Accuracy:** Proportion of correctly predicted sites (both positive and negative).
  $$\text{Accuracy}=\frac{\text{TP}+\text{TN}}{\text{TP}+\text{TN}+\text{FP}+\text{FN}}$$
- **Precision:** Proportion of positive predictions that are correct.
  $$\text{Precision}=\frac{\text{TP}}{\text{TP}+\text{FP}}$$
- **Recall:** Proportion of actual positives correctly identified.
  $$\text{Recall}=\frac{\text{TP}}{\text{TP}+\text{FN}}$$
- **F1 score:** Harmonic mean of precision and recall, indicating balance.
  $$F1=2\cdot \frac{\text{Precision}\cdot \text{Recall}}{\text{Precision}+\text{Recall}}$$
- **Specificity:** Proportion of negatives correctly identified.
  $$\text{Specificity}=\frac{\text{TN}}{\text{TN}+\text{FP}}$$
- **MCC:** Comprehensive measure of classification quality accounting for all confusion matrix components.
  $$\text{MCC}=\frac{\left(\text{TP}\cdot \text{TN})-(\text{FP}\cdot \text{FN}\right)}{\sqrt{\left(\text{TP}+\text{FP})(\text{TP}+\text{FN})(\text{TN}+\text{FP})(\text{TN}+\text{FN}\right)}}$$
- **AUROC and AUPRC:** Area under the ROC curve measures model discrimination, while the Area under the PR curve emphasizes the trade-off between precision and recall.

### 2.2 Features

In our study, we explored multiple features inspired by existing works to improve succinylated lysine residue prediction. The authors of LMSuccSite (Pokharel *et al.* 2022) utilized a combination of word embedding and language model embeddings for residue prediction. A window of amino acids, centered at the target site, is passed through Keras’s (Charles 2013) embedding layer to generate the word embedding. The idea of encoding the amino acid window in this way was first used in DeepSuccinylSite (Thapa *et al.* 2020), where the authors performed experiments with different window sizes (ranging from 11 to 41). The optimal window size from their experiments was 33 (16-K-16), which was later verified in LMSuccSite (Pokharel *et al.* 2022) by similar experiments. Therefore, we too have used a window size of 33. This window denoted as


$$W=[a_{-16},\ldots ,a_{0},\ldots ,a_{+16}]$$

where $a_{0}$ represents the target lysine and $a_{i}$ represents each surrounding amino acid that is transformed into a dense, continuous vector of fixed dimension by Keras’s embedding layer. So, the Keras embedding layer, defined as a function $E:A\to R^{d}$ where A is the set of amino acids (20 canonical amino acids plus additional one for a missing amino acid), transforms each amino acid $a_{i}\in W$ into a *d*-dimensional vector. In our case, d=21, meaning each amino acid is represented by a 21-dimensional embedding vector. The output of the embedding layer for a given amino acid window *W* can be expressed as:


$$E(W)=[E(a_{-16}),\ldots ,E(a_{0}),\ldots ,E(a_{+16})]\in R^{33\times 21}$$

where $E(a_{i})$ is the learned embedding vector for amino acid $a_{i}$, and the embedding layer learns the optimal values for *E* during training. The loss function *L* for the entire model is minimized through backpropagation, updating the embedding matrix *E* as part of this process. This allows *E* to adapt so that the embeddings capture the information necessary for effective succinylation prediction. Thus, the embedding layer should learn representations that enhance succinylation prediction by capturing each amino acid’s specific features and their relation to neighboring residues through training.

Additionally, we considered embeddings from four different language models: ProtT5, ESM-650M, ESM-3B, and PTM-Mamba. ProtT5 (Elnaggar *et al.* 2022), originally called ProtT5-XL-UniRef50, is pretrained using span-generation and teacher-forcing methods in a self-supervised manner on the UniRef50 (Suzek *et al.* 2007) dataset, which has over 45 million protein sequences. It has a 24-layer encoder-decoder design with about 2.8 billion parameters, based on Google’s T5-3B model (Raffel *et al.* 2020). The model can learn contextual links within protein sequences with the aid of its span-generation process, which yields a 1024-dimensional embedding that incorporates both local and global sequence properties. The ESM-650M model is a more recent PLM based on a transformer architecture that was created especially for protein sequences. It comes from the evolutionary scale modeling (ESM) (Lin *et al.* 2023) family, which was also pretrained in a self-supervised manner. By learning to predict masked amino acids inside the sequence, it captures structural and evolutionary insights and generates a 1280-dimensional embedding. Similarly, we used a larger model from the ESM family, the ESM-3B model, with almost 3 billion parameters. The same masked language modeling method as ESM-650M is used to train this model, which has a 2560-dimensional embedding, allowing it to capture far more intricate and distant interactions in protein sequences. We have also incorporated embeddings from PTM-Mamba (Peng 2024), a recently proposed PLM tailored for PTM prediction. PTM-Mamba builds on the Mamba architecture by introducing bidirectional gated Mamba blocks that efficiently capture long-range dependencies in protein sequences while remaining computationally efficient. Trained on a PTM-aware dataset, this model produces a 768-dimensional embedding per residue that encodes both structural and modification-specific context. Its PTM-awareness makes it particularly relevant for succinylation prediction.

Furthermore, we included position-specific scoring matrix (PSSM) as another feature group. PSSM profiles, obtained via sequence alignment against protein databases, provide evolutionary conservation information by highlighting the residues that are crucial for protein function or stability. We hypothesized that PSSM would enhance the performance of the word embedding by emphasizing conserved regions. The feature size of the PSSM matrix was 20.

### 2.3 Feature selection

We incorporated different feature selection methods to select the best feature group from the above-mentioned ones. Because the feature groups had an uneven number of features, we first reduced the dimension of each group to ten using principal component analysis (PCA) (Abdi and Williams 2010) to properly compare the feature importance of each feature group. Then we used the minimum redundancy maximum relevance (mRMR) (Peng *et al.* 2005) technique on the training set to understand which feature group has the most impact in predicting succinylated lysine residues. We also utilized feature importance ranking from XGBoost (eXtreme Gradient Boosting). In this case, we ranked the feature groups based on the cumulative feature importance rankings extracted from XGBoost classifier.

**Figure 1.**
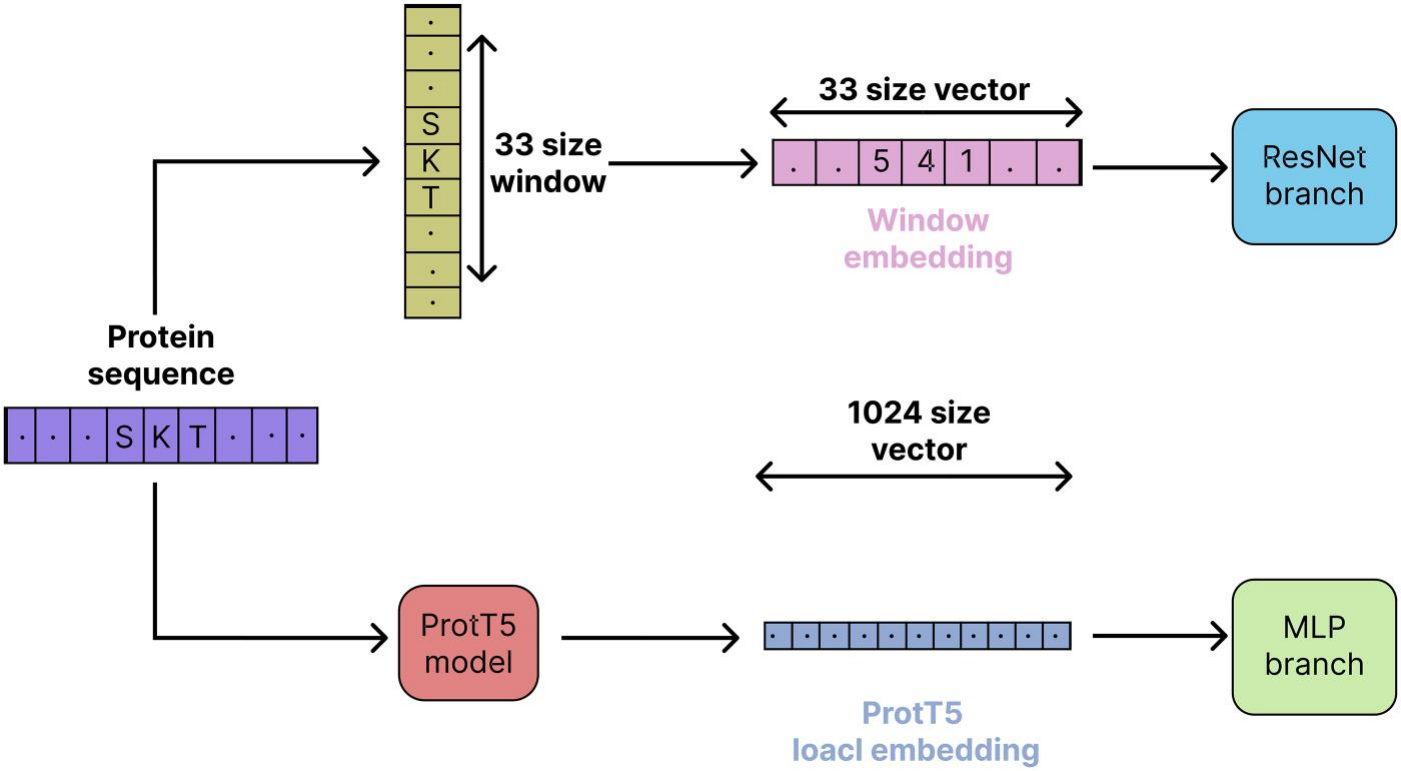
Feature extraction from sequence and protein language model.

In both experiments, most features were selected from word embedding and ProtT5 embedding. Therefore, similar to LMSuccSite (Pokharel *et al.* 2022), we decided to use these feature groups as the final feature set for our model. The complete feature extraction pipeline of the selected features is shown in Figure 1.

### 2.4 Model selection

For our model, we used two separate architectures for word embedding and ProtT5 embedding, as they represent different types of contextual information of the target site.

#### 2.4.1 Word embedding model selection

Because the word embedding is essentially sequential data, we only considered the architectures that are designed to capture sequential relationships more effectively, such as recurrent neural networks (RNN), bidirectional long short-term memory networks (BiLSTM), feedforward neural networks (FNN), and one-dimensional convolutional neural networks (Conv1D). However, in the development of LMSuccSIte (Pokharel *et al.* 2022), two-dimensional convolutional neural networks (Conv2D) were used in their word embedding module. This did not seem to be a good choice because the embedding dimension (like a two-dimensional grid in images) is not as relevant as the sequential dimension (one-dimensional) in an amino acid sequence representation. Each amino acid’s position in a protein sequence has a specific meaning, and applying two-dimensional convolutions could mix up the relationships in a way that might not represent the sequential nature of the sequence window.

However, we still included Conv2D in our model comparison process along with Conv1D and 2 other variations of Conv1D (inception and residual connection). We created an imbalanced validation dataset by splitting the training dataset into a 1:9 ratio conserving the ratio of positive and negative data points in both splits. We named the 10% training set as the validation dataset. As the training set was balanced, the validation set was also balanced. To make it imbalanced, we randomly removed 90% positive data points from the validation set and created 10 imbalanced validation sets. Finally, we trained all seven models using the word embedding and tested their performances on the validation sets. From the results, we chose the basic Conv1D architecture and its two variations as all of them significantly outperformed other deep learning architectures. The Inception and residual variations performed better than the basic Conv1D model in most cases.

#### 2.4.2 ProtT5 embedding model selection

For the ProtT5 embedding part, we compared the results of different traditional machine learning (ML) and complex deep learning (DL) architectures in a similar way. Based on the performance results, MLP was selected for the ProtT5 embedding due to its superiority across multiple performance metrics.

### 2.5 Short description of the models

Based on our preliminary analysis, we decided to further experiment with three different ensemble architectures for succinylated lysine residue prediction: (i) a one-dimensional convolutional network combined with MLP, (ii) an inception module combined with MLP, and (iii) a Residual network combined with MLP. These models were referred to as ConvLysEmbed, InceptLysEmbed, and ResLysEmbed, respectively. Each of the three models has two different branches for separately processing word embedding features and language model embedding (ProtT5) features.

#### 2.5.1 One-dimensional convolutional network (word embedding branch of ConvLysEmbed)

The ConvLysEmbed model incorporates a simple one-dimensional convolution branch that starts with the keras’s embedding layer, which is essentially the key part of word embedding generation. It maps each amino acid in the 33-length amino acid sequence into a 21-dimensional space. The embedding layer is followed by two 1D convolutional layers with 32 and 64 filters, respectively. Each layer has 21 input channels, each representing a column in the embedding of an amino acid. To reduce the embedding dimension, each Conv1D layer is followed by a max-pooling operation. After flattening the output from the convolutional layers, the branch ends with a fully connected dense layer of 32 units, followed by a dropout of 30% to prevent overfitting.

#### 2.5.2 Inception module (word embedding branch of InceptLysEmbed)

The InceptLysEmbed model utilizes an inception module to capture patterns at different scales from the output of the embedding layer. The inception module consists of multiple one-dimensional convolutions in parallel. Each one-dimensional convolutional layer in the Inception module performs a convolution operation with a different kernel size *k* (e.g. k=1,3,5,7,9,11) to capture different patterns. For each kernel size *k*, we have:

$$y_{k}=\text{ReLU}\left(W_{k}*x+b_{k}\right)$$

where:

-
$W_{k}\in R^{k\times D\times F}$ is the weight tensor for the convolution with kernel size *k*, feature dimension *D* and *F* filters,
* denotes the one-dimensional convolution operation, and
-
$b_{k}\in R^{F}$ is the bias term.

The output $y_{k}$ from each branch has shape (L,F), where *F* is the number of filters and *L* is the input sequence length (window size). The output from the embedding layer is simultaneously passed through all these layers. Additionally, a max-pooling branch is combined with one-dimensional convolution with a 1×1 kernel. The outputs from all these branches are then concatenated:

$$y_{\text{concat}}=\text{Concat}(y_{1},y_{3},y_{5},y_{7},y_{9},y_{11},y_{\text{pool}})$$

The resulting output $y_{\text{concat}}$ with shape (L,7F) is processed through max-pooling and flattening operations. Finally, the branch ends with a fully connected dense layer of 32 units similar to the ConvLysEmbed model.

#### 2.5.3 Residual network (word embedding branch of ResLysEmbed)

The ResLysEmbed model employs a residual network (ResNet) architecture which helps preserve important information throughout the convolutional process. Each residual block consists of two Conv1D layers (each with kernel size 3) and a skip connection, which concatenates the input of the block back to the output. For a given residual block, let the input to the block be $x\in R^{L\times D}$, where *L* is the length of the input sequence and *D* is the number of features (e.g. embedding dimension). The output after applying two convolution operations and the skip connection is given by:


$$\text{Block}\;\text{Output}=\text{ReLU}(\text{Concat}(W_{2}*\text{ReLU}(W_{1}*x+b_{1})+b_{2}),x)$$

Where:

-
$W_{1}\in R^{3\times D\times F_{1}}$ is the weight tensor for the first Conv1D layer with $F_{1}$ filters,
-
$W_{2}\in R^{3\times F_{1}\times F_{2}}$ is the weight tensor for the second Conv1D layer with $F_{2}$ filters,
-
$b_{1}\in R^{F_{1}}$ and $b_{2}\in R^{F_{2}}$ are the bias terms for each layer,
* denotes the one-dimensional convolution operation,
- Concat denotes the skip connection that concatenates the input × with the output of the block along the feature dimension.

The ReLU activation function is applied to the output of each convolutional layer. The output from the embedding layer is passed through the first residual block with $F_{1}=32$ filters which is followed by a max-pooling operation. Then again, the output is passed through the second residual block with $F_{2}=64$ filters, which is also followed by a max-pooling operation. Finally, the branch ends similarly to the above-mentioned ones with a flattening operation followed by a dense layer and a dropout of 30%.

#### 2.5.4 MLP branch for ProtT5 embeddings

The MLP branch handles the ProtT5 embedding of the target lysine site, and its architecture is the same across all three separate models. It simply passes the 1024-dimensional embedding through a fully connected layer with 32 units and ReLU activation, followed by a dropout layer. We used a simple architecture of the MLP branch to ensure that the ProtT5 embeddings were effectively processed without overshadowing the contributions of the word embedding branch. The reason for choosing a simple architecture is further justified in a later section.

#### 2.5.5 Model output and loss function

The outputs from the word embedding branch (either Conv1D, or Inception, or ResNet) and the MLP branch are concatenated into a single vector:

$$y_{\text{concat}}=\text{Concat}(y_{\text{word}\;\text{embedding}},y_{\text{MLP}})$$

This concatenated vector is then passed through a fully connected layer followed by a dropout of 30%. From there the model finally makes a binary prediction using the sigmoid function. The model is trained using the Adam optimizer(Kingma 2014) with a learning rate of 0.0001 and uses binary cross-entropy (BCE) loss to measure the prediction error:

$$L=-\frac{1}{N}\sum_{i=1}^{N}\left[y_{i}\;\text{log}({\hat{y}}_{i})+(1-y_{i})\;\text{log}(1-{\hat{y}}_{i})\right]$$

Where:

- *N* is the number of samples,
-
$y_{i}$ is the true label for sample *i*,
-
${\hat{y}}_{i}$ is the predicted probability for sample *i*.

#### 2.5.6 Training and comparing the results of the models

For training the models, we had two options: (i) training the entire model end-to-end and (ii) training the word embedding and ProtT5 branches separately, and then training the combined model, freezing the branches. We used the end-to-end training approach rather than the branched approach used in LMSuccSite (Pokharel *et al.* 2022). This approach was taken to ensure that every learning step of the model is based on the final loss and does not become constrained by the frozen representations of the branching approach (Glasmachers 2017). We also believe that the embedding layer should be trained with the final model so that it can create optimal representations of the amino acids for the final model. On the contrary, training the branches separately and then freezing the layers for the final training might result in a sub-optimal feature encoding in the word embedding branch. As shown later in Section 3.7.1, end-to-end training can in fact result in a better learning process and improved performance in this scenario.

**Figure 2.**
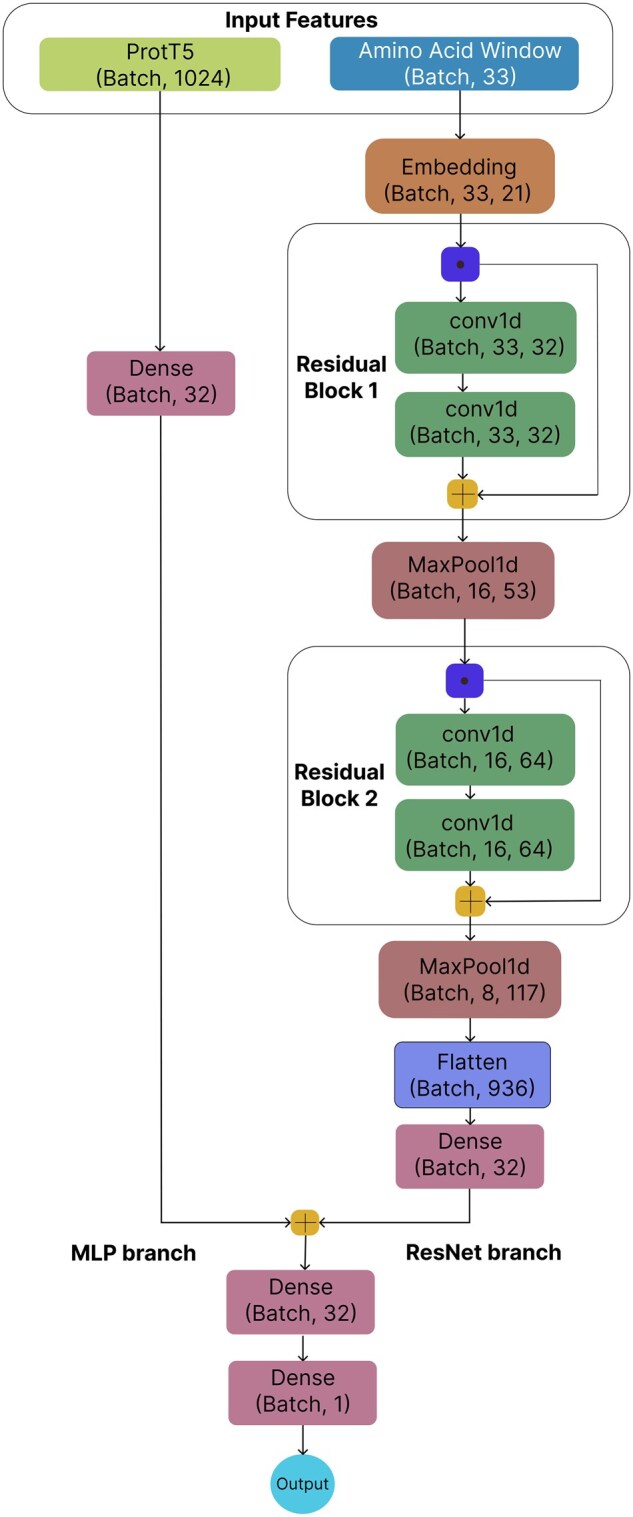
A visual representation of the ResLysEmbed model architecture.

We tested the aforementioned three models using 10-fold cross-validation on the training set and also compared them using ROC and PR curves. The results demonstrate that the ResLysEmbed model is the most effective for succinylated lysine residue prediction, outperforming the ConvLysEmbed and InceptLysEmbed models across all evaluation metrics. Subsequently, we chose ResLysEmbed as our final model for the succinylated lysine residue prediction task. The framework is shown in Fig. 2.

### 2.6 SHAP analysis for interpretability

We used SHAP (Lundberg and Lee 2017) analysis on the ResNet branch of our ResLysEmbed model to understand how each individual residue within the 33-length window centered around the target site affects succinylation prediction of the model. This interpretability analysis allowed us to identify which residues have a significant role in the model’s prediction, providing insight into the significance of local sequence context and the spatial proximity of residues around the target site.

To ensure the robustness of our analysis, we used the dbPTM1815 dataset (Chung *et al.* 2025). Since structural information was essential for our interpretability study, we sourced protein structures from the AlphaFold database (Jumper *et al.* 2021). Out of 1815 proteins in the dbPTM1815 dataset, 1794 had corresponding AlphaFold entries, yielding a total of 3897 datapoints for SHAP analysis. Then we used the SHAP library’s KernelExplainer to calculate the SHAP values as it works especially well with models that do not have inherent differentiability, such as our ResLysEmbed’s ResNet branch. KernelExplainer approximates SHAP values by comparing model predictions for different combinations of input features (in this case residues) included and excluded in each subset. The SHAP value assigned to each residue represents its average contribution to the model’s prediction across these combinations, reflecting how the presence or absence of each residue affects the outcome. Finally, to interpret the SHAP values assigned to each residue more effectively, we implemented two ordering systems, which are explained in Section 3.5.

To ensure consistency and reproducibility, all computed SHAP values and calculated distance metrics were stored in serialized files (pickle format) and are available in our GitHub repository.

## 3 Results

### 3.1 Feature selection results

**Figure 3.**
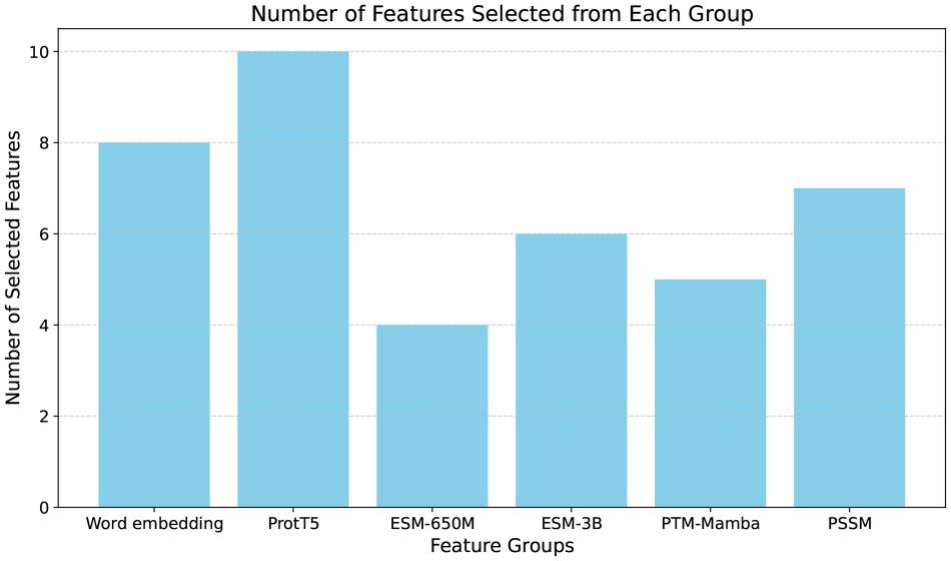
Feature selection using mRMR.

**Figure 4.**
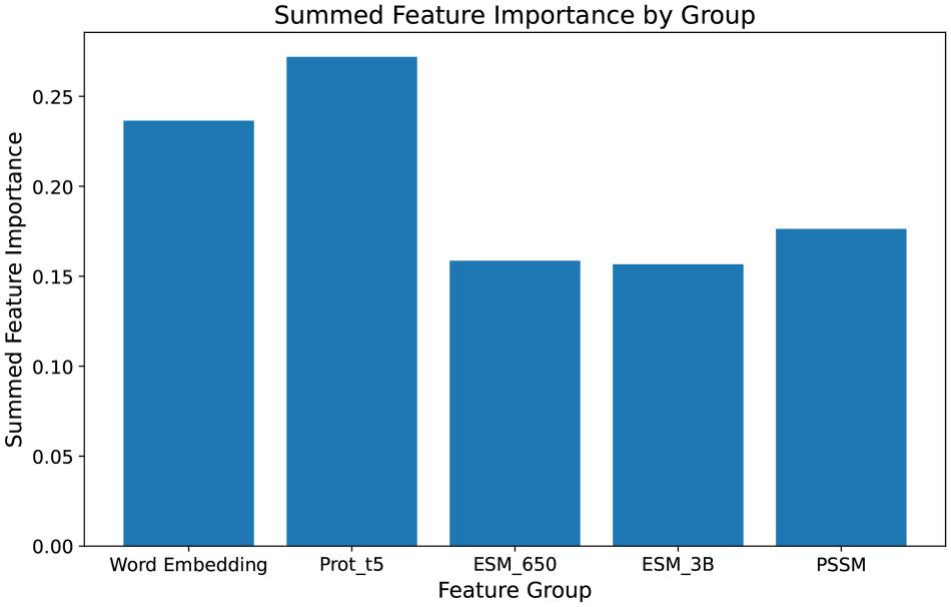
Feature selection using summed feature importance of each feature group.

Figures 3 and 4 shows the results of feature selection. After using PCA (Abdi and Williams 2010) to reduce each feature group to 10 features, we used mRMR to select the top 40 features. The results from Fig. 3 depict how many features were selected from each group after using mRMR on the training set. It is clear from the figure that most of the features were selected from word embedding and ProtT5 embedding. Similarly, Fig. 4 plots the summed importance of the six feature groups while training an XGBoost model on the training set. Here, we can also see that among the six feature groups, ProtT5 and word embedding features showed the highest importance. Based on the results, we concluded that ProtT5 embedding performed much better in terms of succinylated lysine residue prediction than PTM-Mamba, ESM-650M, or ESM-3B. It should also be noted that word embedding, despite not being as sophisticated as a language model embedding, still held substantial relevance for this task, highlighting its importance alongside more advanced embeddings.

### 3.2 Model selection results

#### 3.2.1 Performance comparison of different deep learning models on word embedding

To select the best model for word embedding processing, seven deep learning architectures were experimented with: RNN, BiLSTM, FNN, Conv1D, Conv2D, inception (Conv1D-based), and residual connection (Conv1D-based). The models were trained on the 90% training set and evaluated on 10 randomly imbalanced validation sets.

**Figure 5.**
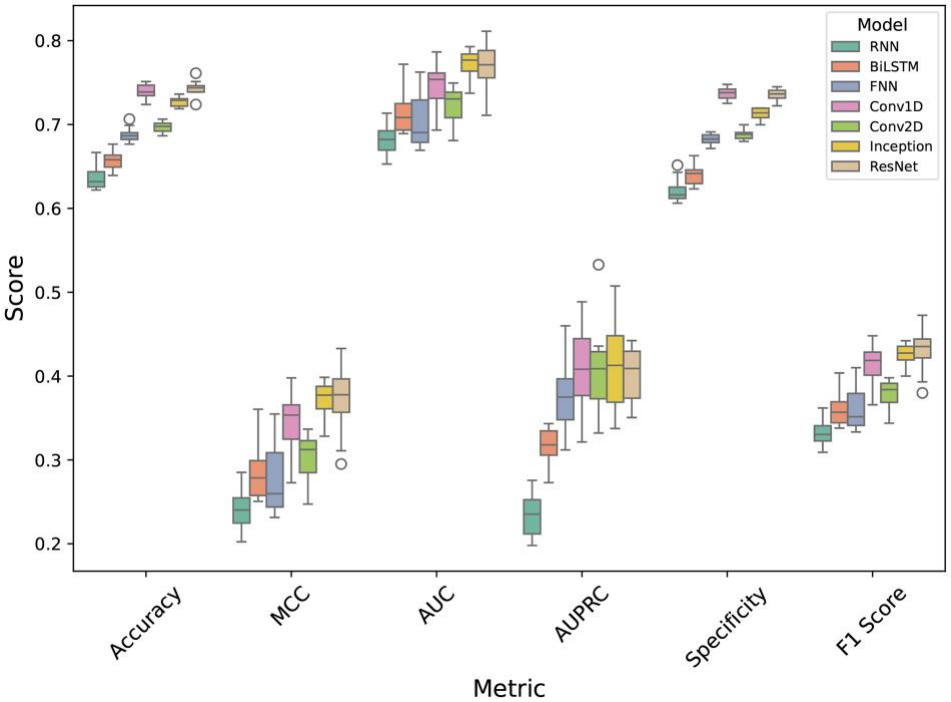
Performance comparison of different deep learning architectures on word embedding. Models were trained on the 90% training set and tested on 10 randomly imbalanced validation sets.

From the results of Fig. 5, we can see that Conv1D architecture and its two variations (Inception and ResNet) significantly outperformed other deep learning architectures. Additionally, RNN and BiLSTM, while having advantages in handling sequential data, they underperformed compared to Conv1D in this scenario. It should also be noted that although the ResNet model performed slightly better in different metrics than the basic Conv1D and Inception, its improvements were marginal. That is why we decided to further experiment with all 3 Conv1D-based architectures for the final model.

Interestingly, the results of Fig. 5 also show that Conv2D does not offer any advantage over Conv1D in this scenario. This confirmed our earlier speculation that applying 2D convolutions could mix relationships between the spatial and sequential dimensions, and that could in turn result in an undesired representation of the sequential nature of the amino acid window.

#### 3.2.2 Performance comparison of different traditional ML and DL models on ProtT5 embedding

**Figure 6.**
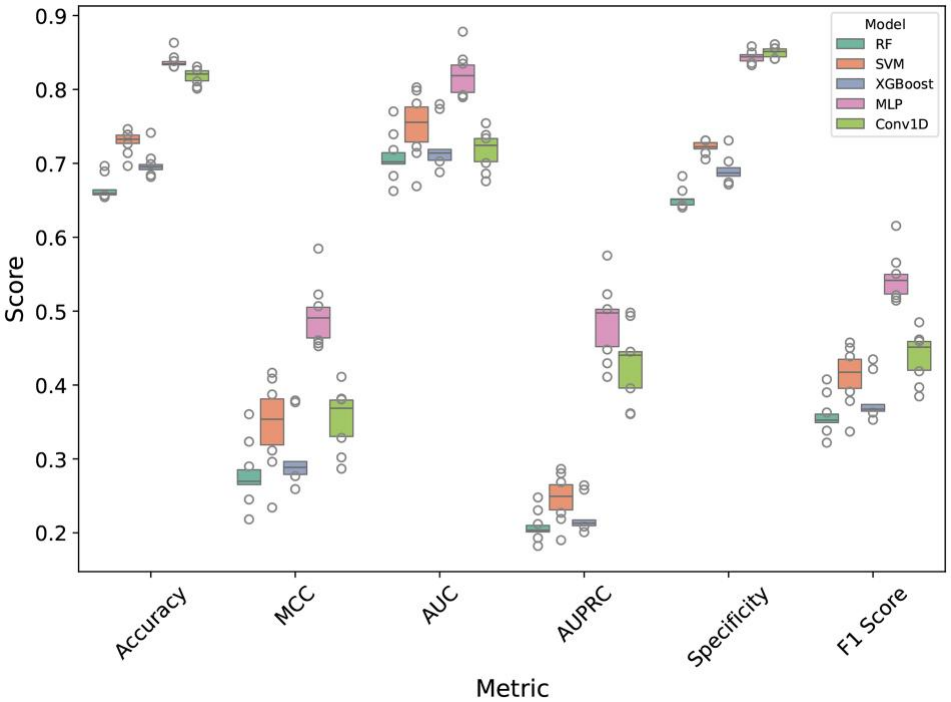
Performance comparison of different models on ProtT5 embedding. The models were trained on the 90% training set and evaluated on 10 randomly imbalanced validation sets.

For selecting the best model for ProtT5 embedding, we tried different ML and DL models namely, Random forest classifier (RF), SVM, Extreme gradient boosting (XGBoost), MLP, and Conv1D. From the results of Fig. 6, we can see that MLP achieved the highest performance across almost all metrics. This suggests that the MLP architecture is more effective for the task of capturing proper information from language model embeddings.

#### 3.2.3 Performance comparison of ConvLysEmbed, InceptLysEmbed, and ResLysEmbed

We compared the performance of ConvLysEmbed, InceptLysEmbed, and ResLysEmbed using 10-fold cross-validation on the training set.

**Figure 7.**
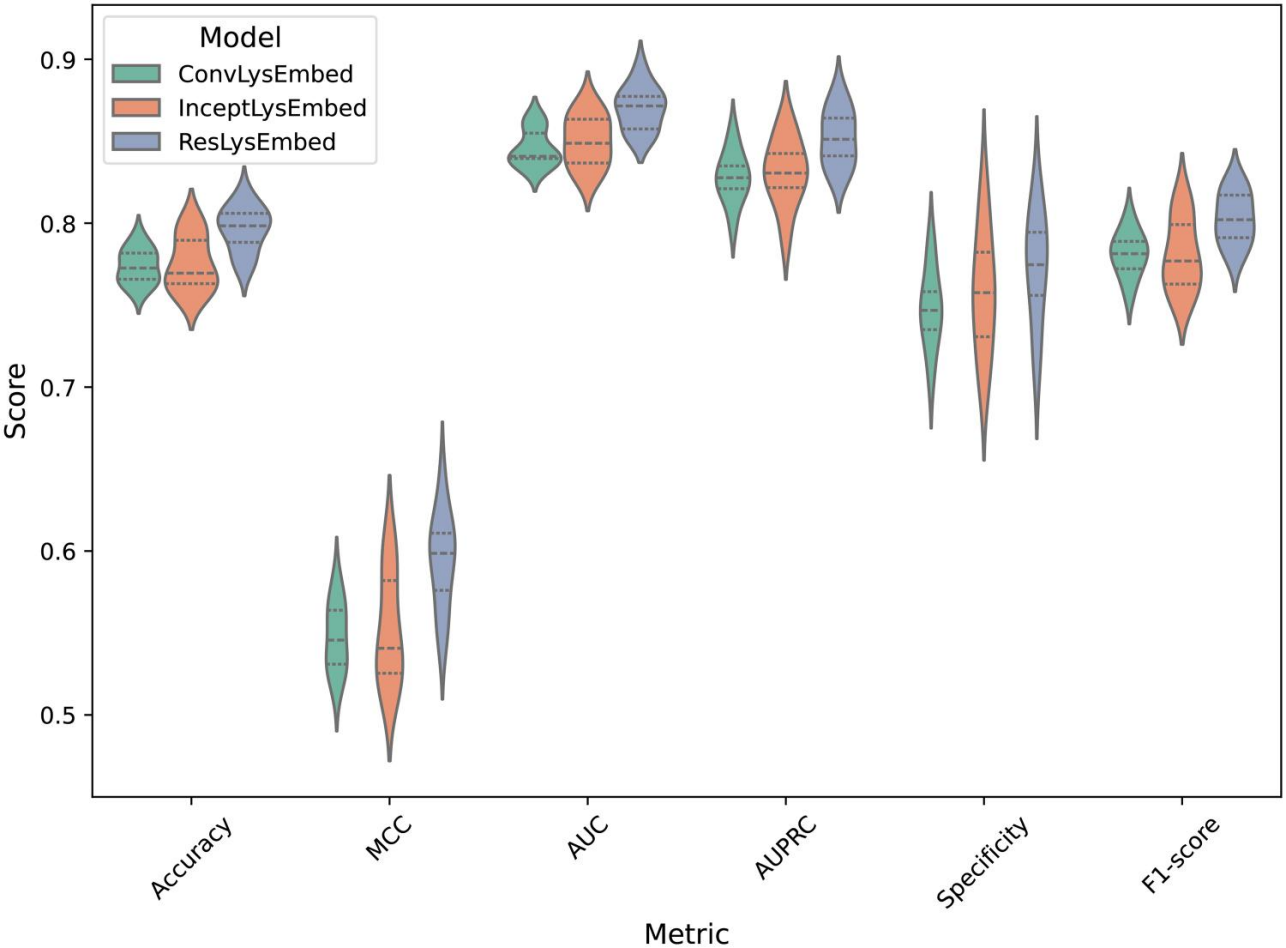
Ten-fold CV performance comparison of ConvLysEmbed, InceptLysEmbed, and ResLysEmbed on the training set.

**Figure 8.**
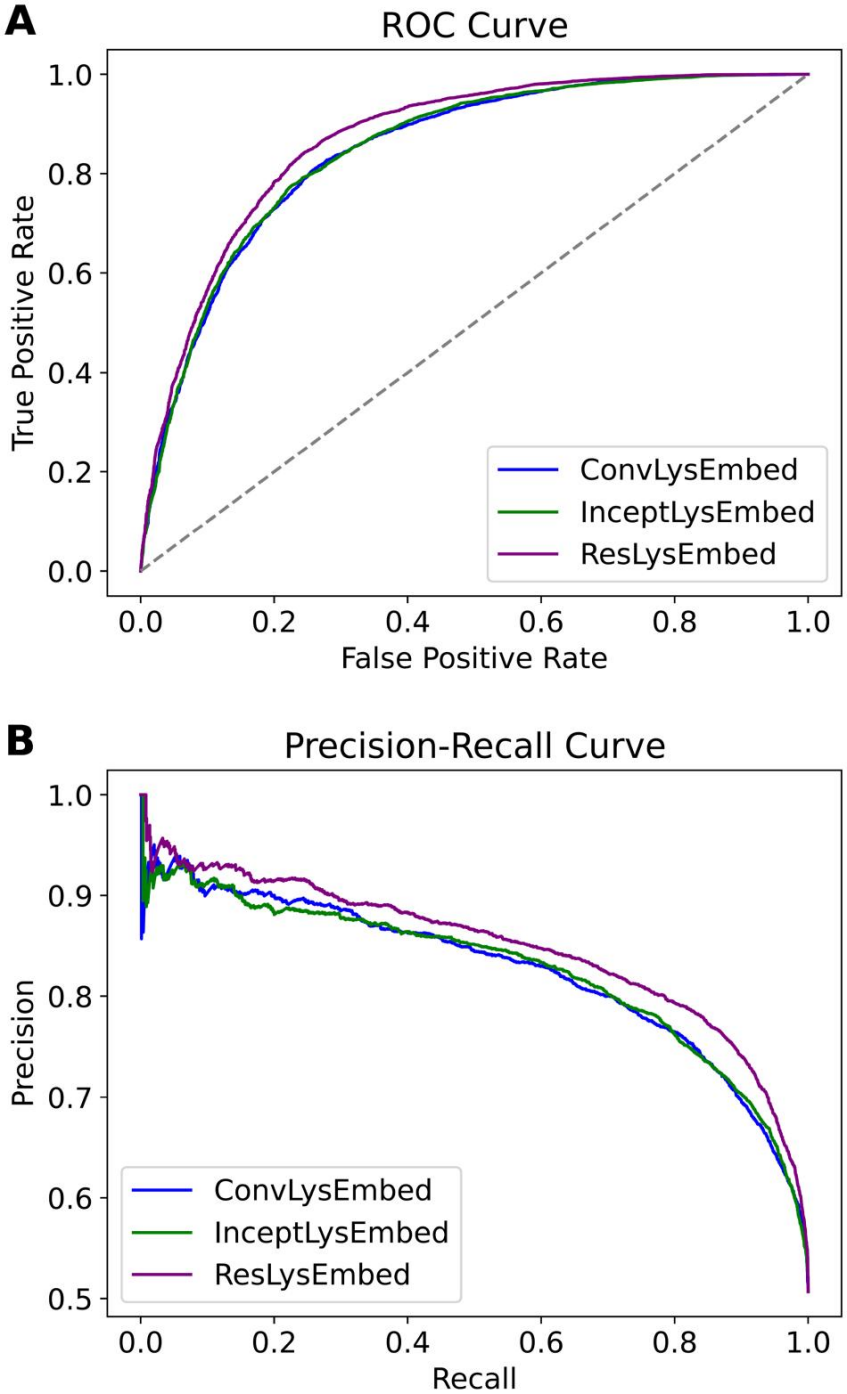
Ten-fold CV results. (A) Receiver operating characteristic (ROC) curves and (B) Precision–recall (PR) curves with area under the curve (AUC) values for ConvLysEmbed, InceptLysEmbed, and ResLysEmbed on the training dataset.

From Fig. 7 and Table 2, we could see that the ResLysEmbed model outperformed the other two models across all metrics. On the other hand, InceptLysEmbed performed similarly to ConvLysEmbed, with only slight improvements in some metrics. Figure 8 visualizes the performance differences of these models using the ROC and PR curves. In Fig. 8, the ROC plot shows that ResLysEmbed consistently has a higher true positive rate at different false-positive rates, resulting in its higher AUC score of 0.80 compared to the 0.77 of both ConvLysEmbed and InceptLysEmbed. Similarly, the PR curve in Fig. 8 shows that ResLysEmbed maintains the highest AUPRC score of 0.85.

From the results, we concluded that the ResLysEmbed was the most effective model at predicting succinylated lysine residues. While ConvLysEmbed and InceptLysEmbed were competent models, their performances were slightly inferior to ResLysEmbed across all metrics. The outcome of this comparison suggests that the residual branch paired up well with the MLP branch for succinylated lysine residue prediction.

#### 3.2.4 Benchmarking ResLysEmbed against baseline models

**Figure 9.**
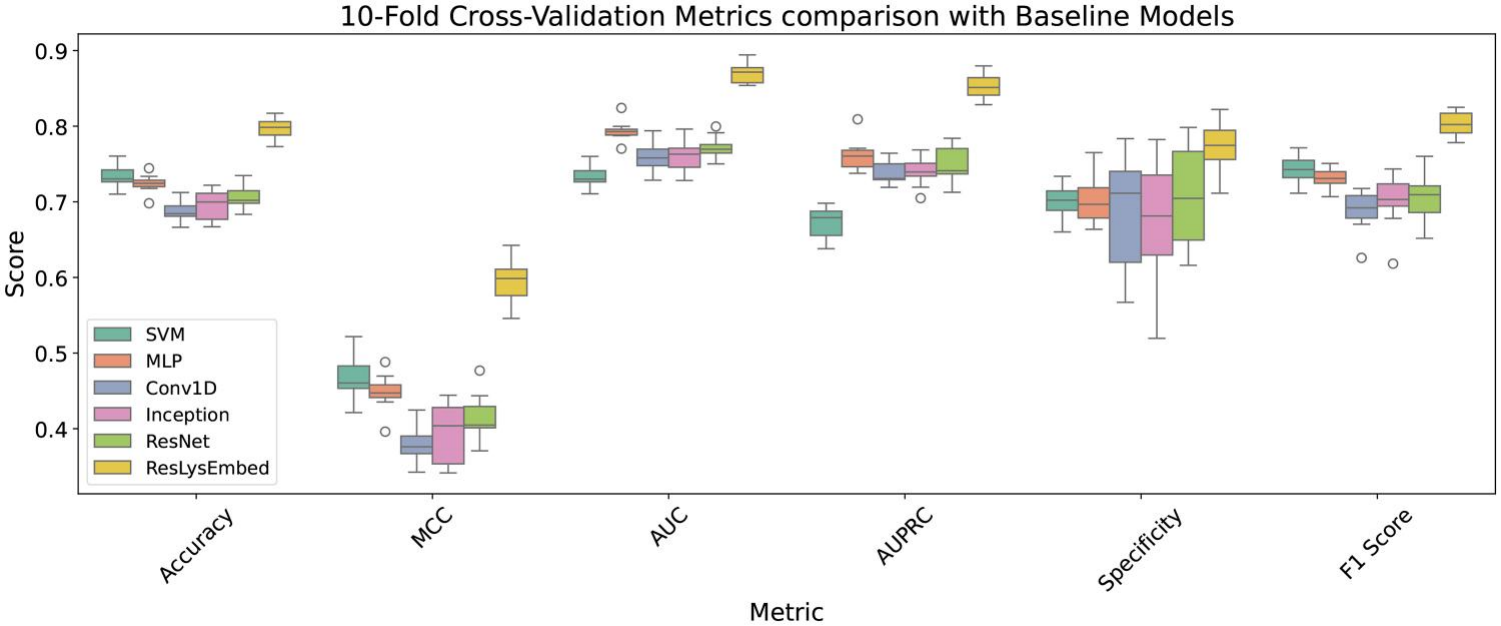
Ten-fold CV performance comparison of ResLysEmbed against baseline models on the training set.

To evaluate the effectiveness of ResLysEmbed beyond our internal architectures, we conducted a benchmark against the baseline models we used earlier. We have included a comparison with SVM, MLP, Conv1D, inception, and ResNet because these performed relatively better in our earlier model selection analysis as shown in Figs 5 and 6. Each model was trained and evaluated using 10-fold cross-validation under the same data conditions and preprocessing pipelines as ResLysEmbed. As shown in Fig. 9, ResLysEmbed consistently outperforms the baseline models across all evaluation metrics. This further reinforces the suitability of ResLysEmbed for the lysine succinylation prediction task, not only compared to hybrid architectures, but also when benchmarked against standard machine learning and deep learning approaches.

### 3.3 Performance comparison of PLM embeddings

To validate our feature selection findings further and assess the predictive power of individual PLM embeddings, we performed a 10-fold cross-validation using the ResLysEmbed model with each of the four embeddings—ProtT5, PTM-Mamba, ESM-650M, and ESM-3B on the training dataset. For this experiment, the model architecture and training setup were kept constant, and only the local embedding corresponding to the target lysine residue (from each respective PLM) was changed for each of the models.

**Figure 10.**
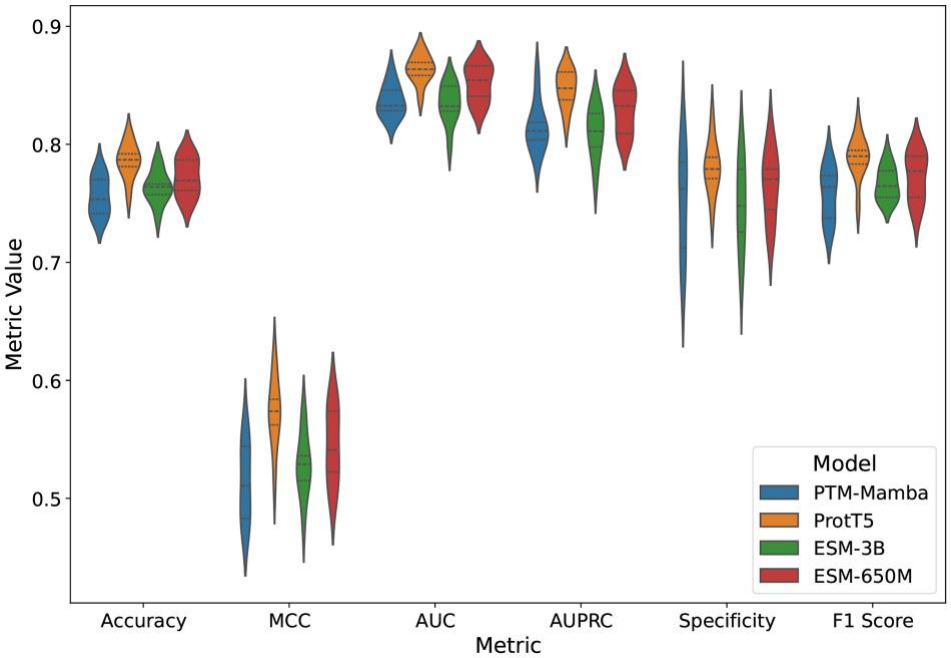
Ten-fold CV performance comparison of ResLysEmbed on training set using different PLM embeddings.

**Figure 11.**
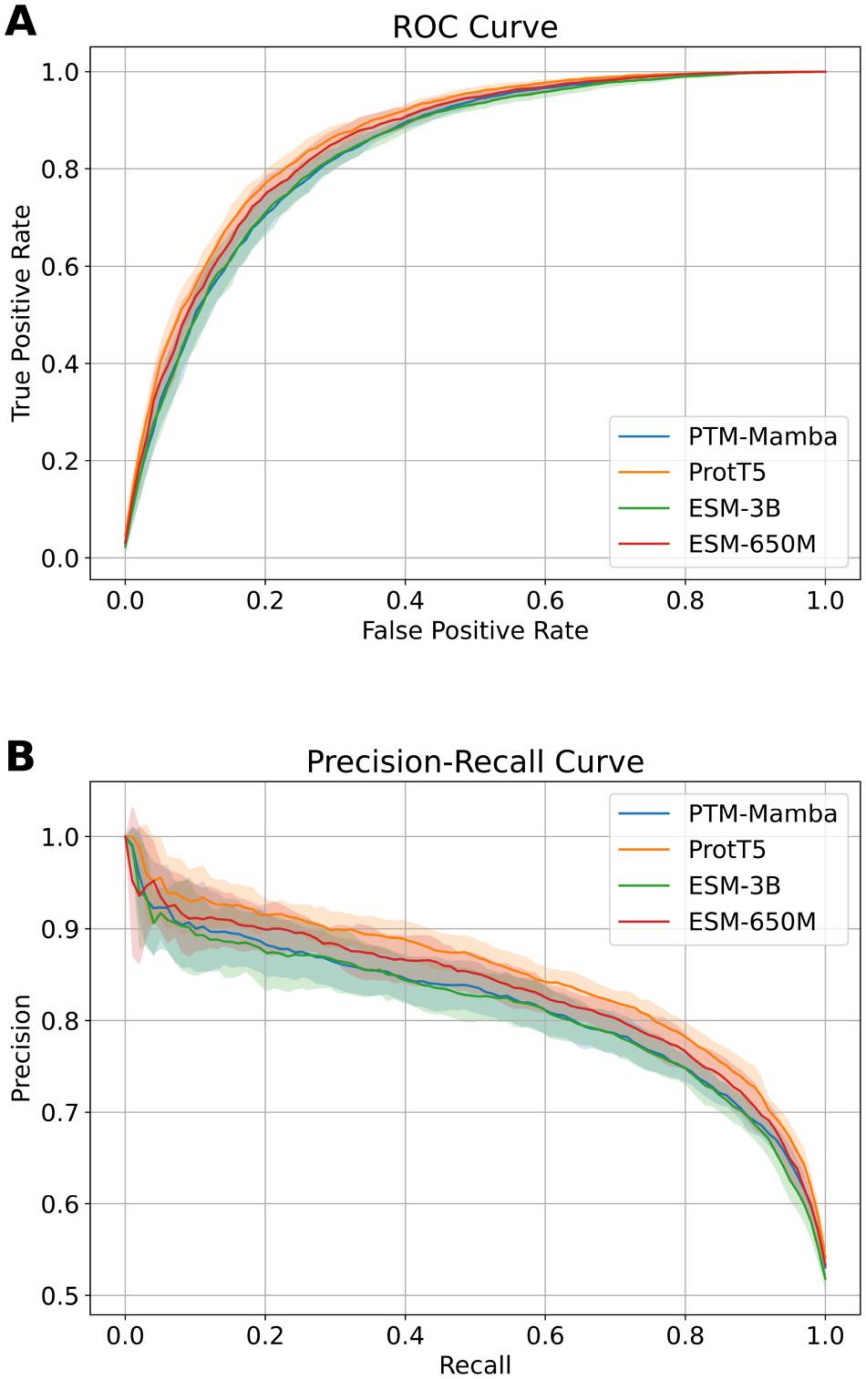
(A) ROC and (B) PR curves from 10-fold CV on the training set for ResLysEmbed using different PLM embeddings.

From Table 3 and Fig. 10, it is evident that ProtT5 outperformed the other embeddings across all metrics. PTM-Mamba, although specifically designed for PTM tasks, performed slightly worse than ProtT5 but relatively close to both ESM variants. The ROC and PR curves (Fig. 11) also confirm the superior performance of ProtT5. These results validate our earlier feature selection findings and support the use of ProtT5 embeddings in the final ResLysEmbed model.

**Table 1.** Overview of the dataset used for training and testing.

Dataset type	Positive (succinylated)	Negative (non-succinylated)	Number of proteins
Training dataset	4750	50 565	2192
Training dataset (after balancing)	4750	4750	2192
Benchmark independent test set	254	2977	124
dbPTM1815 dataset	1901	2309	1815

**Table 2.** Ten-fold CV performance comparison of ConvLysEmbed, InceptLysEmbed, and ResLysEmbed on the training set.^a^

Model	Accuracy	MCC	AUROC	AUPRC	F1
ConvLysEmbed	0.7736 ± 0.0100	0.5476 ± 0.0200	0.7732 ± 0.0100	0.8281 ± 0.0149	0.7811 ± 0.0131
InceptLysEmbed	0.7742 ± 0.0110	0.5487 ± 0.0225	0.7740 ± 0.0109	0.8329 ± 0.0151	0.7805 ± 0.0129
ResLysEmbed	**0.7965 ± 0.0130**	**0.5941 ± 0.0273**	**0.7960 ± 0.0133**	**0.8521 ± 0.0165**	**0.8028 ± 0.0151**

a The scores are represented in mean ± SD format and the boldfaced values indicate the best performance for each evaluation metric.

**Table 3.** Ten-fold CV performance of ResLysEmbed on the training set using different PLM embeddings.^a^

Embedding	Accuracy	MCC	AUROC	AUPRC	F1 score
PTM-Mamba	0.7643 ± 0.0137	0.5297 ± 0.0269	0.8368 ± 0.0091	0.8126 ± 0.0123	0.7655 ± 0.0164
ProtT5	**0.7864 ± 0.0153**	**0.5733 ± 0.0304**	**0.8632 ± 0.0144**	**0.8440 ± 0.0193**	**0.7896 ± 0.0183**
ESM-3B	0.7580 ± 0.0119	0.5180 ± 0.0247	0.8319 ± 0.0152	0.8035 ± 0.0225	0.7624 ± 0.0146
ESM-650M	0.7729 ± 0.0164	0.5468 ± 0.0325	0.8526 ± 0.0147	0.8294 ± 0.0180	0.7744 ± 0.0163

a The scores are represented in mean ± SD format and the boldfaced values indicate the best performance for each evaluation metric.

### 3.4 Comparison of ResLysEmbed with other predictors

In this section, we compared the performance of our proposed ResLysEmbed model with other state-of-the-art models for succinylated lysine residue prediction. For comparison, we had chosen the (CBL + BLC + CBL_BLC)-E model from Ahmed *et al.* (2023) (their best performing model), pSuc-EDBAM (Jia *et al.* 2022), PTM-CMGMS (Li *et al.* 2024), LMSuccSite (Pokharel *et al.* 2022), and a newly published method, KD_MultiSucc (Tran *et al.* 2025). Since LMSuccSite had not reported all the standard performance metrics. Additionally, the saved model from their GitHub repository could not be loaded. We tried contacting the authors via email regarding these issues, but did not receive any response. Therefore, we had to reimplement their work based on the provided methodology and hyperparameter values.

Table 4 represents the key performance metrics including accuracy, MCC, AUC, AUPRC, precision, recall, and F1 score of different models along with ResLysEmbed on the independent test set. It should be noted that the same training set from Table 1 was used to develop these models, and the independent test set from Table 1 was used to evaluate them. Along with our reproduced results of LMSuccSite (Pokharel *et al.* 2022) we have also included the performance metrics from their paper. From the results, it is evident that the ResLysEmbed model outperforms existing methods in all metrics and achieves a significant improvement compared to the next best predictor, LMSuccSite.

**Table 4.** Performance comparison of ResLysEmbed with other predictors on the independent test set.^a^

Model	Accuracy	MCC	AUROC	AUPRC	Precision	Recall	Specificity	F1 score
LMSuccSite^b^	0.79	0.36	–	–	–	0.79	0.79	–
LMSuccSite (retrained)	0.7626	0.3345	0.7808	0.3155	0.2209	0.8024	0.7591	0.3464
pSuc-EDBAM^c^	0.7446	0.3088	0.8357	0.3077	0.2041	0.7875	0.7409	0.3242
PTM-CMGMS^d^	–	0.3072	0.8306	0.3024	–	–	–	–
(CBL + BLC + CBL_BLC)-E^e^	0.696	0.271	–	–	–	0.791	0.787	–
KD_MultiSucc^f^	0.7846	0.2863	0.8346	0.2950	0.2152	0.6601	0.7952	0.3246
ResLysEmbed	**0.8053**	**0.3893**	**0.8733**	**0.3482**	**0.2624**	**0.8182**	**0.8042**	**0.3973**

a Except for LMSuccSite (retrained), all the other results were taken from the respective papers and the boldfaced values indicate the best performance for each evaluation metric.

b Taken from Pokharel *et al.* (2022).

c Taken from Jia *et al.* (2022).

d Taken from Li *et al.* (2024).

e Taken from Ahmed *et al.* (2023).

f Taken from Tran *et al.* (2025).

To further assess our model’s generalizability, we evaluated ResLysEmbed on the dbPTM1815 dataset. Due to the unavailability of trained models, feature extractors, or reproducible pipelines for many of the competing methods, we were only able to evaluate ResLysEmbed, LMSuccSite, pSuc-EDBAM, and KD_MultiSucc on this dataset. From the results recorded in Table 5, it is evident that ResLysEmbed is the best among the lot, securing the best value for all the performance metrics but for specificity. KD_MultiSucc has the best specificity with ResLysEmbed holding the second position. However, KD_MultiSucc’s good specificity comes at a significant cost of recall value—its recall is the worst among the predictors.

**Table 5.** Performance comparison of ResLysEmbed with other predictors on the latest dbPTM1815 dataset.^a^

Model	Accuracy	MCC	AUROC	AUPRC	Precision	Recall	Specificity	F1 score
LMSuccSite	0.7050	0.4003	0.7671	0.7037	0.7097	0.5865	0.8025	0.6423
pSuc-EDBAM	0.6943	0.3781	0.7851	0.7208	0.6965	0.5723	0.7947	0.6284
KD_MultiSucc	0.6809	0.3579	0.7793	0.7114	0.7263	0.4842	**0.8465**	0.5811
ResLysEmbed	**0.7264**	**0.4444**	**0.7889**	**0.7233**	**0.7313**	**0.6228**	0.8116	**0.6727**

a The boldfaced values indicate the best performance for each evaluation metric.

**Figure 12.**
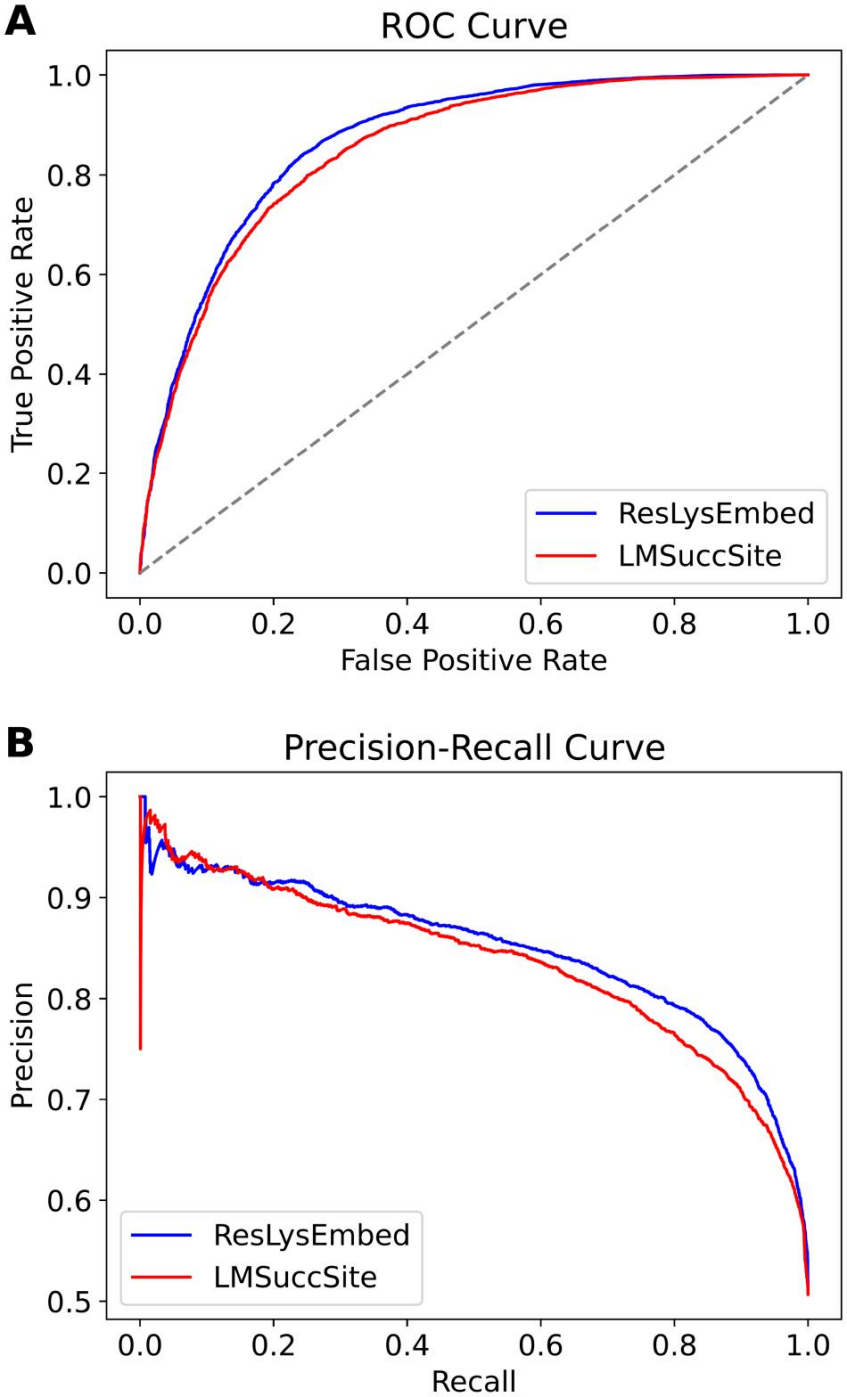
(A) ROC curve and (B) PR curves for ResLysEmbed and LMSuccSite from the 10-fold CV on the training Set.

**Figure 13.**
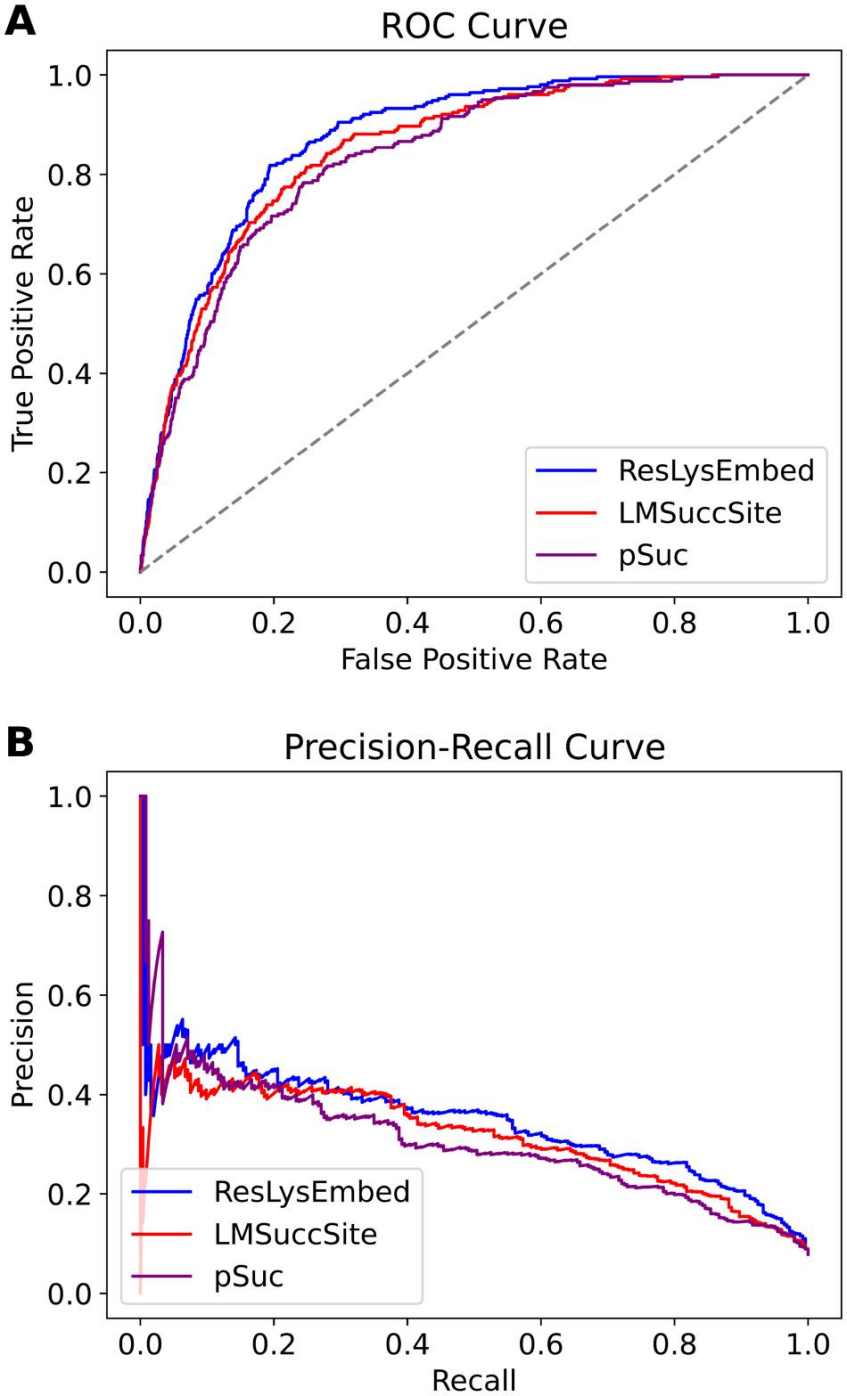
(A) ROC curve and (B) PR curves for ResLysEmbed, LMSuccSite, and pSuc-EDBAM on the independent test set.

We also compared the performance of ResLysEmbed against LMSuccSite (Pokharel *et al.* 2022) using the ROC and PR plots for the 10-fold cross-validation on the training dataset. Then we also compared it against both LMSuccSite (Pokharel *et al.* 2022) and pSuc-EBDAM (Jia *et al.* 2022) on the independent test set. Figures 12 and 13 illustrate the ROC and PR plots for the 10-fold cross-validation results and the independent test results, respectively. As reflected in the higher AUC and AUPRC scores in both ROC and PR plots, the ResLysEmbed model demonstrates better performance in classification compared to both LMSuccSite and pSuc-EDBAM.

We also evaluated the performance of ResLysEmbed against PTMGPT-2 (Shrestha *et al.* 2024), a recently proposed PROTGPT2-based predictor. While PTMGPT-2 has reported strong performance, its evaluation setup raised some concerns due to significant overlap between its training and test sets. Specifically, upon acquiring the UniProt IDs from the authors, we found that 764 proteins were common between the 5879 training proteins and 979 test proteins. Additional CD-HIT analysis at 30% sequence identity revealed high levels of redundancy, suggesting that the reported results on their test set may be overestimated due to data leakage. To provide an informative comparison, we benchmarked both models on the independent test set from Table 1 as well as on progressively filtered subsets generated by applying sequence identity cutoffs using CD-HIT-2D on the independent test set ranging from 0.5 to 0.8. These filtered subsets are designed to assess how well each model generalizes to non-redundant, unseen sequences, which better reflects real-world prediction scenarios.

While PTMGPT-2 achieves higher accuracy, precision, and F1 score on the full independent test set, the overlap with PTMGPT-2’s training data likely inflates these metrics. To examine this, we performed further evaluations at different sequence identity thresholds between PTMGPT-2’s training set and the independent test set using CD-HIT-2D filtering. The results, shown in Table 6, demonstrate a sharp drop in PTMGPT-2’s performance as sequence similarity to PTMGPT-2’s training set decreases, suggesting that the performance in Table 7 had been inflated due to data leakage from similar sequences in the training set.

**Table 6.** Comparison of PTMGPT-2 and ResLysEmbed under different sequence identity filtering on the independent test set.^a^

Cutoff	Pos/neg	Model	Acc.	MCC	AUROC	AUPRC	Prec.	Recall	Spec.	F1
0.5	4/123	PTMGPT-2	**0.9528**	0.2256	0.6128	0.0861	**0.2500**	0.2500	**0.9756**	0.2500
		ResLysEmbed	0.8583	**0.3940**	**0.9502**	**0.2984**	0.1818	**1.0000**	0.8537	**0.3077**
0.6	5/190	PTMGPT-2	**0.9282**	0.2168	0.6711	0.0769	**0.1538**	0.4000	**0.9421**	0.2222
		ResLysEmbed	0.8410	**0.3409**	**0.9289**	**0.1947**	0.1389	**1.0000**	0.8368	**0.2439**
0.7	6/227	PTMGPT-2	**0.9099**	0.1496	0.6292	0.0523	0.1053	0.3333	**0.9251**	0.1600
		ResLysEmbed	0.8069	**0.3071**	**0.9159**	**0.1574**	**0.1176**	**1.0000**	0.8018	**0.2105**
0.8	10/323	PTMGPT-2	**0.8739**	0.0521	0.5474	0.0351	0.0556	0.2000	**0.8947**	0.0870
		ResLysEmbed	0.7628	**0.2558**	**0.8556**	**0.1210**	**0.1034**	**0.9000**	0.7585	**0.1856**

a Pos/neg column reports the number of positive and negative samples used in evaluation and the boldfaced values indicate the best performance for each evaluation metric.

**Table 7.** Performance comparison of PTMGPT-2 and ResLysEmbed on the full independent test set (253 positives, 2973 negatives).^a^

Pos/neg	Model	Accuracy	MCC	AUROC	AUPRC	Precision	Recall	Specificity	F1 score
253/2973	PTMGPT-2	**0.9209**	**0.5921**	0.8663	**0.4133**	**0.4963**	0.8016	**0.9310**	**0.6131**
253/2973	ResLysEmbed	0.8053	0.3893	**0.8733**	0.3482	0.2624	**0.8182**	0.8042	0.3973

a The boldfaced values indicate the best performance for each evaluation metric.

As shown in Table 6, although PTMGPT-2 performs strongly on the unfiltered test set, its performance drops significantly as redundant sequences are progressively removed. While the standard practice for redundancy reduction is to apply a 30% sequence identity cutoff, doing so in our case resulted in only a few negative samples, making it impractical to draw statistically meaningful conclusions. So, we gradually relaxed the identity threshold up to 80%. Nevertheless, even at 80%, the data remained sparse (e.g. only 10 positives and 323 negatives). Yet, its performance drops significantly after filtering out redundant sequences, particularly in terms of recall, AUPRC, and F1 score, suggesting that it tends to favor the majority (negative) class and fails to generalize when presented with novel or dissimilar sequences. For example, at the 0.5 sequence identity threshold, PTMGPT-2 achieved only 0.25 recall and 0.0861 AUPRC, while ResLysEmbed achieved 1.00 recall and 0.2984 AUPRC on the same set. We also attempted to evaluate PTMGPT-2 on the dbPTM1815 dataset using the same sequence identity filtering strategy (0.5–0.8) applied earlier. However, for identity thresholds below 0.8, the number of test samples after filtering was extremely small (e.g. only 27 negative samples and no positive samples at the 0.7 threshold). At the 0.8 identity threshold, where barely some positive and negative samples remained (9 positives and 87 negatives), ResLysEmbed achieved an MCC of 0.3440, AUROC of 0.8059, and AUPRC of 0.3719, compared to PTMGPT-2’s MCC of 0.2721, AUROC of 0.6648, and AUPRC of 0.1791. These results demonstrate that ResLysEmbed maintains its effectiveness on non-redundant, unseen sequences, thereby supporting its value beyond what is already available in the field.

#### 3.4.1 Further analysis using t-SNE plots

**Figure 14.**
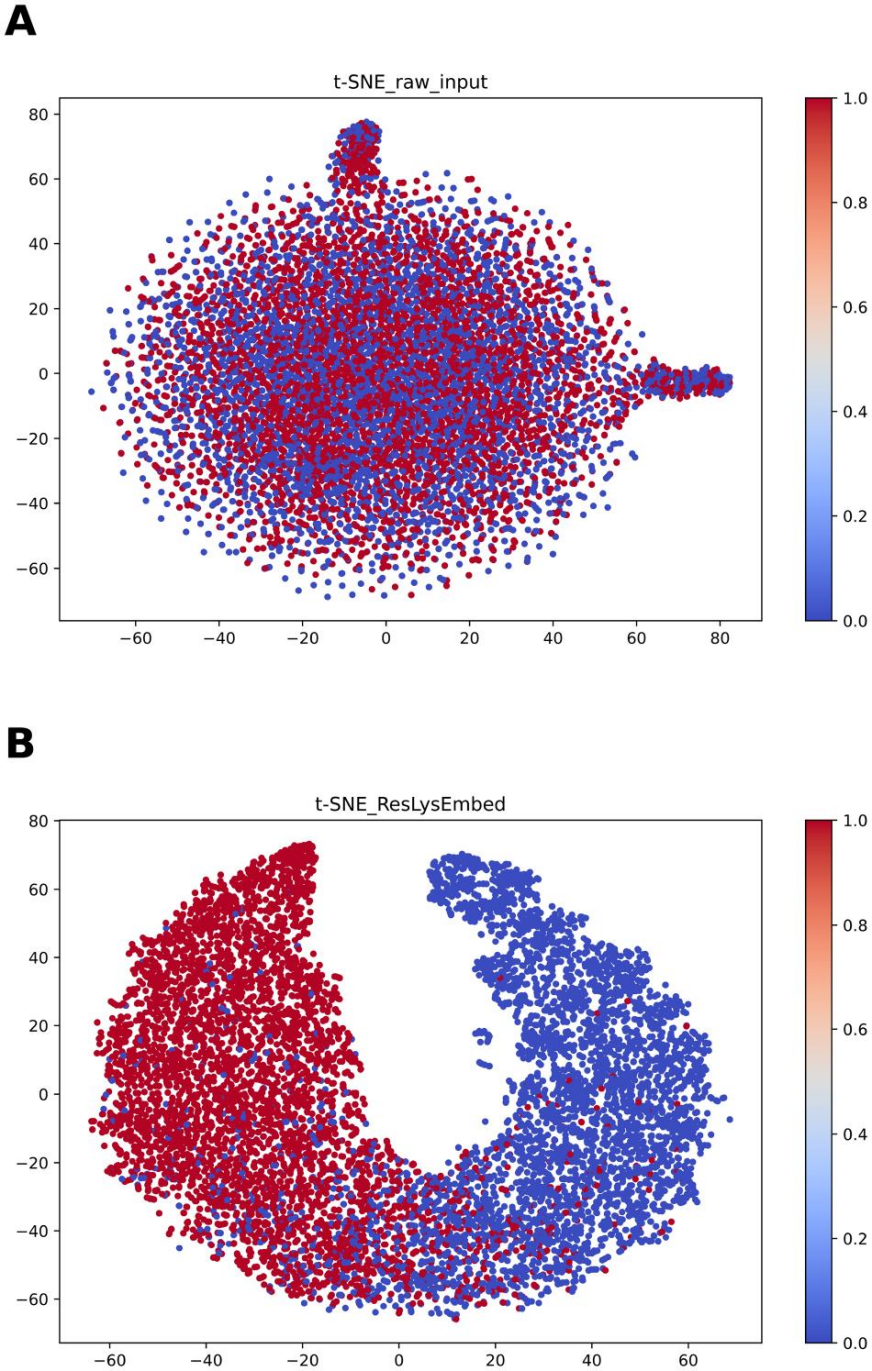
t-SNE visualization of feature representations. (A) Raw input features before training, and (B) features from the final hidden layer after training the ResLysEmbed model.

To get a more visual look at the model’s learning capability, we visualized the high-dimensional embeddings using t-distributed stochastic neighbor embedding (t-SNE) (Van der Maaten and Hinton 2008), as shown in Fig. 14.

The top figure (A) shows the raw input features (from the training dataset) before training. There is no clear separation between the succinylated and non-succinylated lysine residue data. After training the ResLysEmbed model, we plotted the features from the final hidden layer which is shown in the bottom figure (B). Interestingly, it shows significantly improved separation, with almost distinct clusters forming for positive and negative classes, which confirms that the model was able to learn distinctive features from the input data.

### 3.5 SHAP analysis on ResNet branch

In order to gain insights into how each residue from the 33-length window affects our model’s outcome, we performed SHAP (Lundberg and Lee 2017) analysis on the ResNet branch using the dbPTM1815 dataset (Chung *et al.* 2025). We used two different orderings to interpret the SHAP values of each residue in the window: sequence-based ordering and structural distance-based ordering from the target residue.

**Figure 15.**
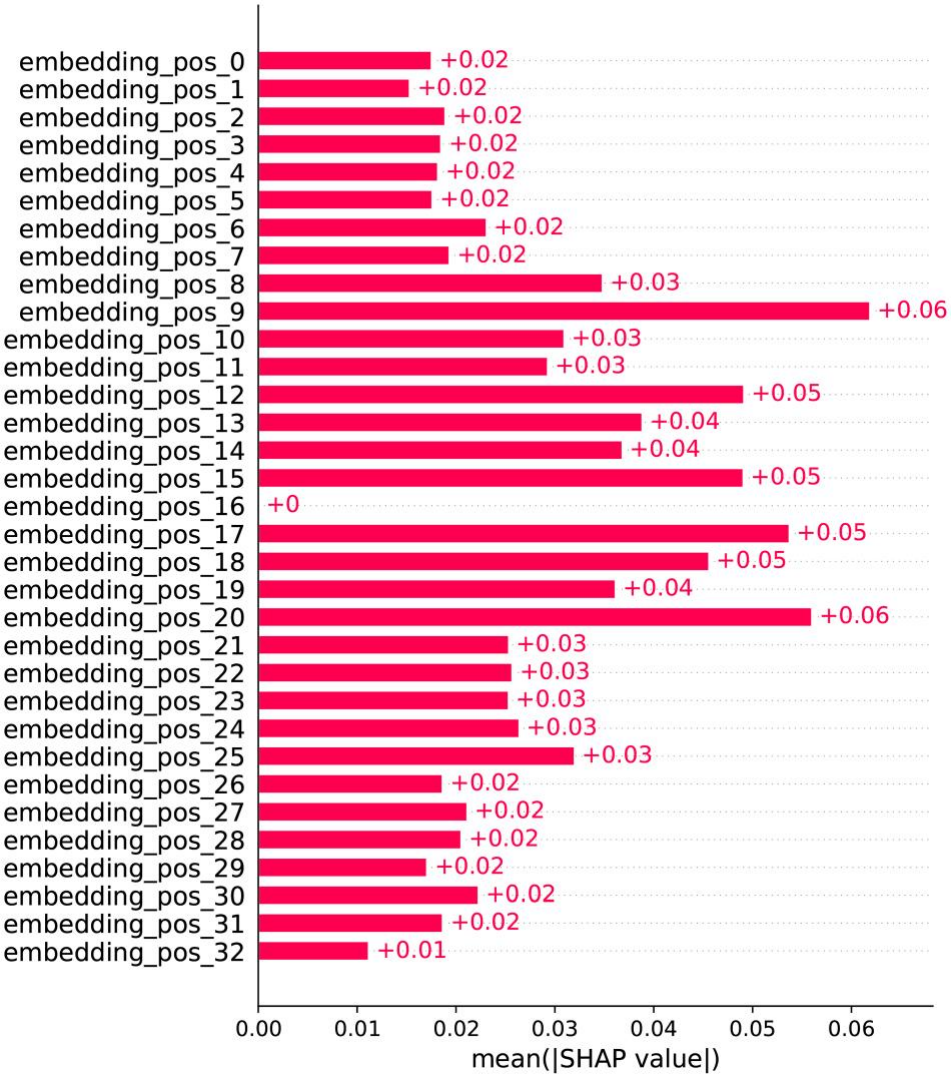
SHAP analysis on ResNet branch using dbPTM1815 dataset—sequence-based plot.

**Figure 16.**
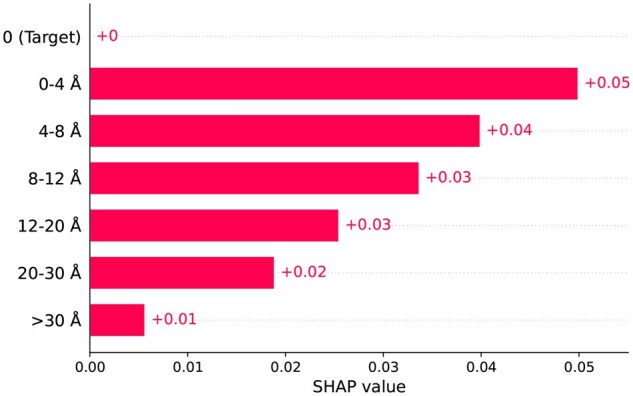
SHAP analysis on ResNet branch using dbPTM1815 dataset—structural distance-based plot.

Figure 15 shows the contribution of each residue in the window to the prediction based on their position in the sequence. Here, the 16th residue is the target residue, and residues from 0 to 15 are upstream and 17 to 32 are downstream in the sequence. From the figure, the first thing that catches the eye is that the target residue at embedding position 16 has 0 contribution to the prediction. This is quite obvious as the target residue is always lysine. However, the important takeaway from the figure is that the contributions of the surrounding residues are quite high around the target site, and as we move further away from the target site in the sequence the contributions of the residues also tend to decrease gradually, albeit there are exceptions—residues at position 9 and 20 have exceptionally high SHAP values. This suggests that a residue’s contribution is not solely dependent on its position in the sequence. For further investigation, we conducted an additional SHAP analysis based on structural distance rather than sequence order. Figure 16 demonstrates the average contribution of residues based on their distance from the target site in the structure. Here, the distance values in the y-axis of the figure are divided into several segments based on their proximity to the target residue. As expected, the target, which is the first one with 0 Å distance, has no contribution to the prediction. However, after the target residue, we can see that the SHAP values decrease as the distance from the target residue increases. Notably, residues within the closest proximity to the target (0–4 Å) exhibit the highest SHAP values, indicating their dominant influence. Beyond this range, the contributions steadily decrease as the distance increases. This pattern illustrates that succinylation is highly influenced by residues that are closer in the protein’s three-dimensional structure, regardless of their position in the sequence.

We emphasize that the aim of this analysis was not to interpret the high-dimensional ProtT5-based features. Instead, our interpretability focus was on understanding which positions in the input sequence contribute most to the model’s decision making based on sequence-derived features. Given the relatively interpretable nature of word embeddings, SHAP provides a meaningful understanding of the residue-level importance learned by the ResNet. Overall, the SHAP analysis demonstrates that ResLysEmbed appears to be able to capture structural cues, despite not being provided with any structural features directly, and exhibits a preference for structurally proximal residues. Further investigation is needed to better understand how such models are able to deduce structural information.

### 3.6 Ablation study: comparison of ResLysEmbed with its individual branches (ResNet and MLP) on two test datasets

To assess the contribution of each individual input stream to the final prediction performance, we compared the full ResLysEmbed model with its two branches trained and evaluated independently: (i) the ResNet-style branch that processes sequence-based word embedding features and (ii) the MLP-based branch that processes the ProtT5-derived local embedding of the target residue. All three configurations were trained on the original training dataset described in Section 2.1.

**Figure 17.**
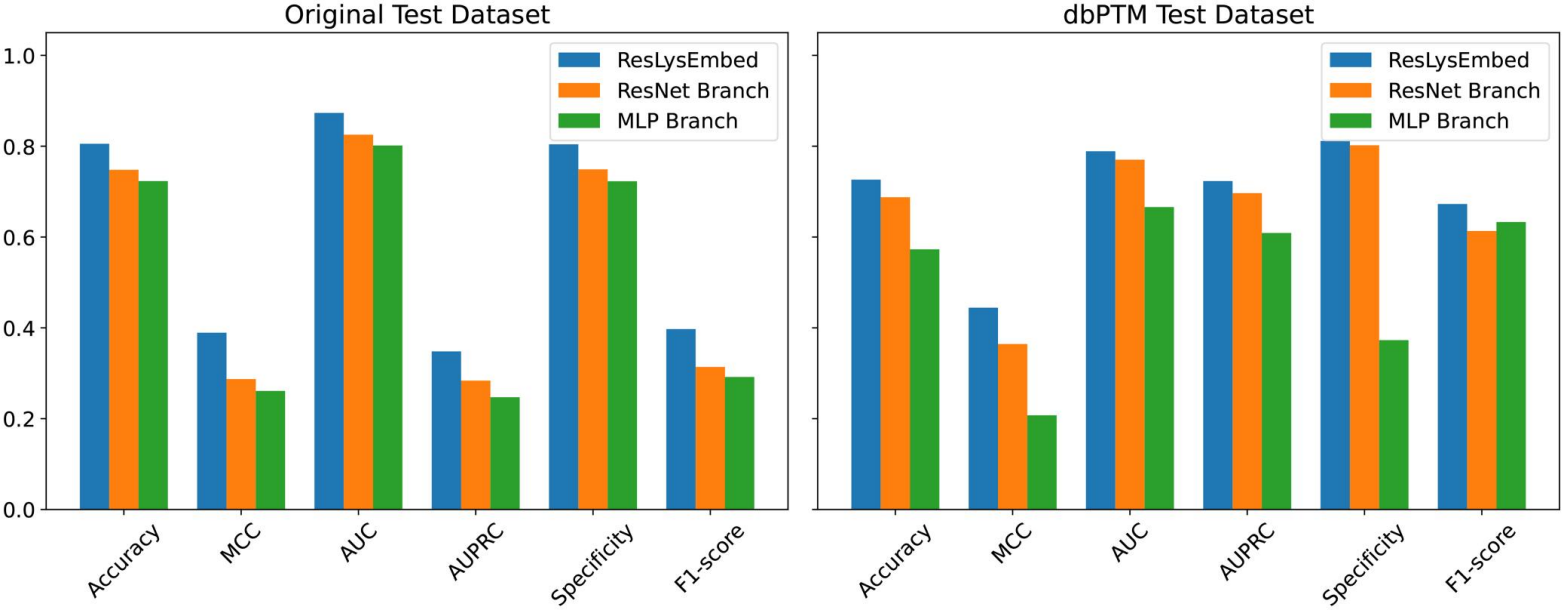
Performance comparison of ResLysEmbed and its two branches (ResNet and MLP)—left: original test dataset, right: dbPTM1815 dataset across six evaluation metrics.

Figure 17 summarizes the evaluation metrics for all three models on both the original test set and the dbPTM1815 dataset. Across both datasets, the full ResLysEmbed model outperforms its individual branches, indicating that combining both sources of information results in a more accurate and robust predictor. While the ResNet and MLP branches each achieve competitive performance individually, their combination in the full model yields a consistent improvement, suggesting that the dual-branch design enhances the model’s overall performance.

### 3.7 Evaluation of training strategies and branch complexity in model performance

#### 3.7.1 Comparison of training the model end-to-end and training the branches separately

In this section, we compare two different training approaches discussed earlier: (i) training the entire model end-to-end without any branches and (ii) training the word embedding and ProtT5 branches separately, and then training the combined model, freezing the branches. Based on the results from Table 8, we observe that the end-to-end trained model outperforms the branched model across almost all metrics. The results demonstrate that training the model holistically is the better approach in this scenario.

**Table 8.** Comparison of ResLysembed performance for end-to-end training and branched training on the validation set.^a^

Model	Accuracy	MCC	AUC	AUPRC	Precision	Recall	Specificity	F1 score
ResLysembed (end-to-end)	**0.8064**	**0.6144**	**0.8803**	**0.8579**	**0.8380**	0.7854	**0.8299**	**0.8109**
ResLysembed (branched)	0.7936	0.5880	0.8735	0.8568	0.7785	**0.8201**	0.7671	0.7987

a The boldfaced values indicate the best performance for each evaluation metric.

#### 3.7.2 Impact of MLP branch complexity on model performance

**Figure 18.**
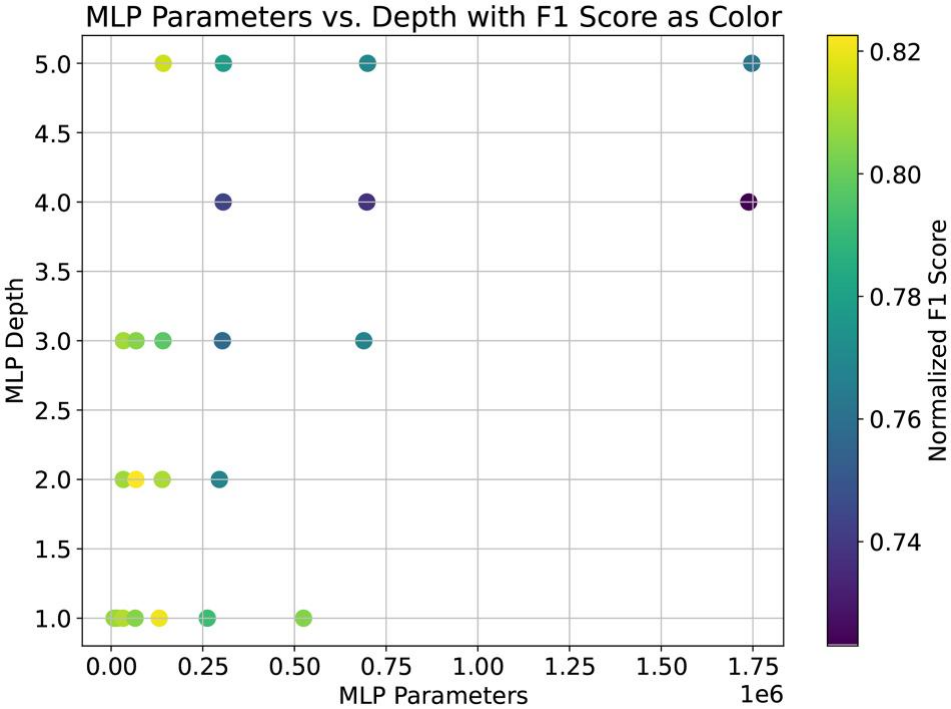
F1 score on the validation dataset *versus* the complexity of the MLP branch.

The MLP branch in our model is responsible for processing the 1024-dimensional local embedding of the target site generated from ProtT5. As shown in Fig. 2, the MLP branch is quite simple in our model. We tried several configurations of the MLP branch, ranging from a very simple architecture (a dense layer with only eight units) to more complex architectures with several layers with a large number of units. Then we tested the ResLysEmbed model with all of these different MLP branches on the validation set. From Fig. 18, we can see that, as the complexity of the MLP branch increases, F1 score initially rises. However, there is a noticeable decline in the metric beyond a certain level of complexity, indicating that an overly complex MLP branch can lead to overfitting and reduce the model’s generalizability. This result validates our choice of the simple architecture for the MLP branch, which also ensures computational efficiency with fewer parameters and faster predictions.

### 3.8 Case study—ResLysEmbed in diverse structural environments

**Figure 19.**
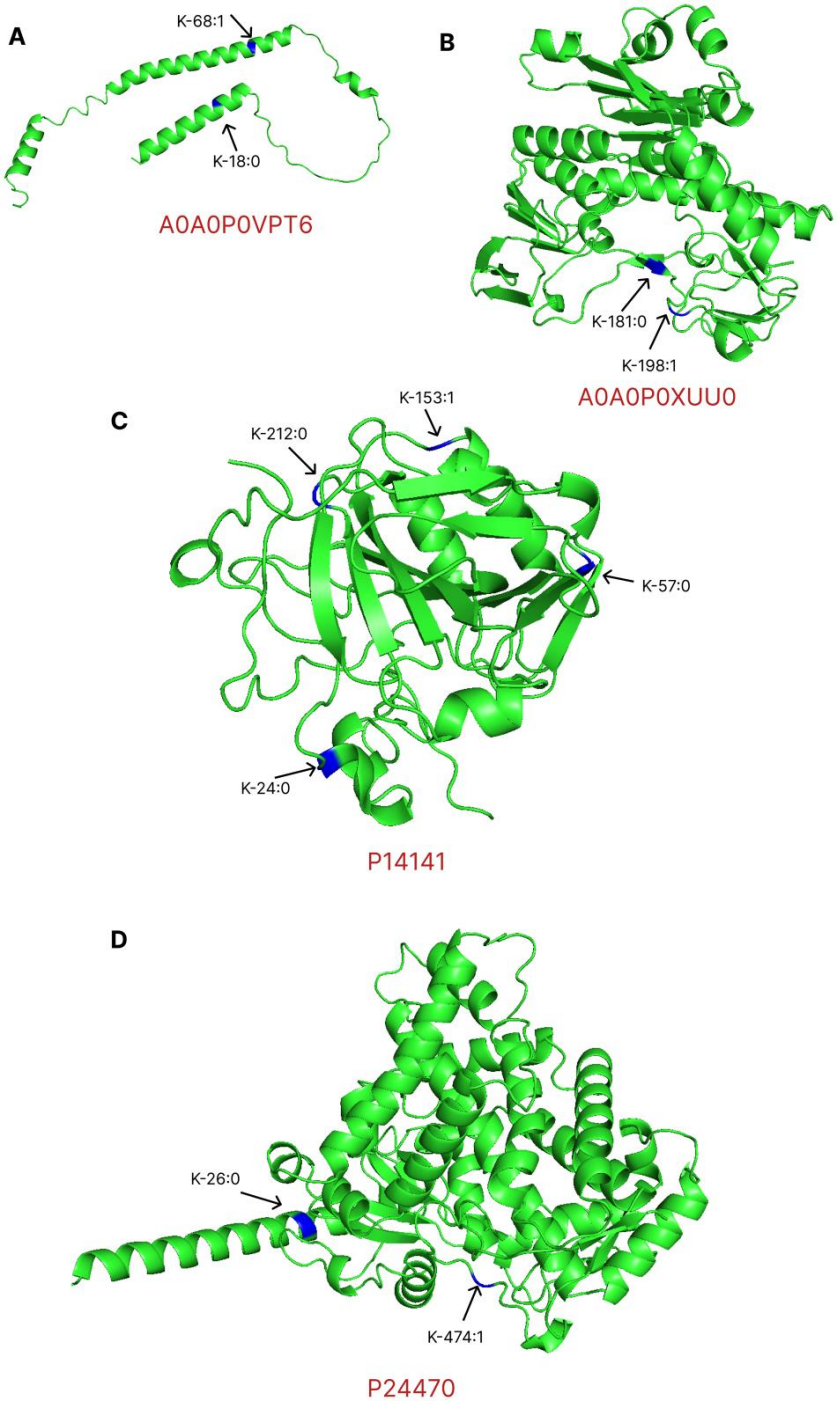
Structural annotation and site visualization for four proteins having Uniprot IDs. (A) A0A0P0VPT6, (B) A0A0P0XUU0, (C) P14141, and (D) P24470. Each subfigure displays the three-dimensional structure of a protein: alpha helices are shown as spiral coils, beta sheets as flat arrows, and connecting regions as wavy lines or random coils. Lysin (K) succinylated and non-succinylated sites are color-coded in blue and labeled in the format K-position: ground truth label, where 1 denotes a succinylated site and 0 a non-succinylated site.

We conducted a case study on four proteins with the UniProt IDs A0A0P0VPT6, A0A0P0XUU0, P14141, and P24470 in Fig. 19. We collected the protein structure predicted by AlphaFold and visualized it using PyMOL (DeLano *et al.* 2002). In all four proteins, all sites were correctly classified by ResLysEmbed. But LMSuccSite and pSuc-EDBAM could not do so. They both failed to correctly predict sites 68 of A0A0P0VPT6, 198 of A0A0P0XUU0, 153 of P14141, and 474 of P24470. From Fig. 19, we can see that site position 68 of A0A0P0VPT6 was in the alpha helices (spiral coils). The site position 198 of A0A0P0XUU0, 153 of P14141, and 474 of P24470 were all in the connecting region (wavy lines or random coils). These observations suggest that although LMSuccSite and pSuc-EDBAM struggled with diverse structural environments, ResLysEmbed consistently made correct predictions, regardless of the structural setting surrounding the sites.

## 4 Discussion

In this study, we have proposed a novel method, ResLysEmbed, which uses a ResNet-based architecture combined with MLP for lysine succinylation site prediction. While our work and LMSuccSite (Pokharel *et al.* 2022) may appear similar at a high level—both utilizing PLM embeddings and word embeddings—ResLysEmbed introduces some key architectural and methodological innovations. Unlike LMSuccSite’s modular architecture with frozen components, ResLysEmbed is trained end-to-end using a ResNet backbone, which enables better feature learning and overall performance. In addition, we systematically assessed alternative feature types, including embeddings from ESM-650M, ESM-3B, and PSSM, and selected ProtT5 and word embeddings based on a rigorous feature selection pipeline involving mRMR and XGBoost importance ranking. We also carefully evaluated multiple architectures and training strategies for each feature type, ultimately selecting the optimal configuration through comparative analysis. To further strengthen our evaluation, we incorporated a more recent and larger benchmark dataset derived from the dbPTM database(Chung *et al.* 2025). In fact, ResLysEmbed outperforms state-of-the-art methods on both datasets, while maintaining a lightweight model with only 93 082 parameters, nearly one-fourth the size of LMSuccSite (361 337 parameters). Furthermore, we conducted SHAP analysis on our model, which revealed the relative importance of residues in the 33-length window based on their sequential position and structural distance from the target site. The results of this analysis highlight the key factors that affect a surrounding residue’s impact on the target site’s succinylation. These results can be valuable in understanding the underlying mechanism of succinylation through further computational or biological analysis.

KD_MultiSucc, a very recent predictor, presented an interesting direction combining multi-teacher knowledge distillation with word embeddings to balance computational efficiency and predictive accuracy. ResLysEmbed achieves better performance than it in two independent test sets without relying on distillation-based frameworks. Our model size (93 082 parameters) is also slightly less than that of KD-MultiSucc (117 802 parameters).

Despite the success of our method, we believe there are areas for improvement and future exploration. For example, we were also unable to use structural features due to resource limitations. Although structural data can provide critical insights, we were only able to get structures for a fraction of the proteins using the AlphaFold database. Generating pdb files for all sequences in the dataset using AlphaFold (Jumper *et al.* 2021) was also infeasible given its computational demands. By using structural features in future studies, we might be able to enhance performance and biological relevance. Furthermore, as more and more PLMs emerge, a more comprehensive analysis of the impact of different PLMs in lysine succinylation site prediction may provide more improvements. In light of emerging methods, we also acknowledge an interesting direction: the use of DeepInsight (Sharma *et al.* 2019) to transform high-dimensional feature vectors (e.g. ProtT5 embeddings) into image-like representations for analysis via two-dimensional CNNs. However, given the semantic nature of protein embeddings and the lack of inherent spatial structure, such transformations may require significant adaptation and may not always provide additional performance or interpretability benefits. Future work could explore the suitability of such representations in the context of protein embedding spaces.

Finally, We hope that ResLysEmbed will serve as a reliable and resource-efficient tool for further research regarding lysine succinylation and related PTMs. The method’s performance on the independent test set shows around 2%–13% improvement in all metrics compared to the next best results. On the newly curated dbPTM1815 dataset, ResLysEmbed again secures the top rank in all core metrics, including MCC (0.4444), F1 score (0.6727), and AUROC (0.7889), further demonstrating its generalizability. Furthermore, the insights gained from our SHAP analysis might be helpful for future computational and biological studies on succinylation mechanisms.

## Data Availability

The dataset used in our study and the Python scripts for reproducing the results are freely available at https://github.com/Sheldor7701/ResLysEmbed.
